# Advanced kidney mass segmentation using VHUCS-Net with protuberance detection network

**DOI:** 10.3389/frai.2026.1716063

**Published:** 2026-02-04

**Authors:** J. Jenifa Sharon, L. Jani Anbarasi

**Affiliations:** School of Computer Science and Engineering, Vellore Institute of Technology, Chennai, India

**Keywords:** abnormality detection, kidney masses segmentation, protuberance detection network, semantic segmentation, transformer enhanced U-Net model, vision transformer, hybrid deep learning, computer-aided diagnosis

## Abstract

**Introduction:**

Accurate segmentation of kidney masses and structure is essential for medical application including diagnosis and treatment. This research proposed the dual track hybrid VHUCS-Net architecture which effectively highlights structural size-shape variants, boundaries and complex structural features in kidney disease.

**Methods:**

Efficient segmentation is achieved by integrating the transformer enhanced U-Net model with the contrast optimized Protuberance Detection Network (PDN) model. The process begins with analysing kidney images using a standard U-Net combined with Vision Transformer attention and a High Resolution Network (HRNet) which capture global dependencies while preserving high resolution features resulting in accurate segmentation of the kidney region. Also, the masked kidney image undergoes processing through a contrast optimized PDN model with multi scale pooling, contrast enhancement, boundary refinement and explicit feature fusion to segment the mass region thereby enhancing mass localization improving border identification and enabling accurate abnormality detection. The resulting features are fused to provide a refined mass segmentation result that exactly identifies the location and structural abnormalities.

**Results:**

The VHUCS-Net model was evaluated using the kidney segmentation dataset achieving an intersection over union score of 0.9441 and a dice coefficient of 0.9712 showing outstanding segmentation precision.

**Discussion:**

These results indicate improved diagnostic efficiency and support clinical decision making by providing more accurate and interpretable segmentation outputs. Moreover, VHUCS-Net is validated with additional publicly available datasets with image mask correspondence, therefore proving the model effectiveness and generalizability across many segmentation tasks. The results highlight the capability of the proposed VHUCS-Net model to enhance diagnostic accuracy and assist clinical decision making through more detailed and interpretable segmentation outcomes.

## 1 Introduction

The kidney is an essential organ responsible for blood filtration, toxin removal, maintenance of electrolyte balance and fluid level regulation (Daniel et al., 2021). These processes are carried out by millions of nephrons which help in maintaining the body internal balance. However, disorders can mainly affect kidney function if they are not immediately recognized and treated. Kidney masses whether malignant or benign required accurate identification and segmentation for best treatment planning. Diagnosis early enhances patient outcomes by enabling proper treatment such as surgical removal, radiation therapy or specialized treatment.

Kidney mass (Lin et al., 2021) develops through several stages requiring multiple diagnostic and treatment approaches. In the initial phase tiny lumps are often small that are usually detected with imaging modalities. As the mass develops structural changes occur requiring continuous monitoring and treatments mainly in advanced stages. Tumors may spread to other organs leading to health risks and decreased chance of survival possibilities if not examined. Exact identification of these stages is important for identifying proper treatment choices to improve the health of patients.

The segmentation of kidney masses is essential for identifying abnormalities, support radiologists and doctors to evaluate kidney mass size, shape and growth for appropriate treatment planning (Zöllner et al., 2021). Various segmentation procedures have been developed to increase accuracy however traditional approaches are time consuming, human error can occur and frequently insufficient for managing the difficulty of kidney masses. The variation in the structural features among individuals shows the limitations of traditional segmentation approaches which are frequently inconsistent, incorrect and inflexible. These challenges highlight the importance for deep learning approaches which provide automation, enhanced precision and robustness in the. Deep learning (Goel et al., 2022) techniques improve segmentation accuracy by effectively segmenting mass boundaries, reducing observer variability and increasing the efficiency of kidney mass detection.

Artificial intelligence (Liu et al., 2023) and deep learning have significantly improved kidney mass segmentation by training models on large datasets providing accurate identification and analysis. This progress is mainly applied to the development and incorporation of multiple deep learning methods. Convolutional neural networks (Hwang et al., 2022) are used for extracting spatial features. Architectures such as U-Net and its variants improve segmentation precision by preserving both local and global contextual information. Moreover, transformer based models like vision transformer use self-attention processes to capture long-range relationships thus improving edge detection. By integrating these methodologies deep learning significantly improves segmentation efficacy allowing the early identification of kidney masses simplifying clinical decision making and improving patient care through more accurate, consistent and efficient analysis.

Contribution of the proposed model:

- The proposed VHUCS-Net model is a dual-track hybrid architecture which integrates a transformer enhanced U-Net with a contrast optimized PDN model for accurate and effective kidney mass segmentation.
- The transformer enhanced U-Net model includes a standard U-Net integrated with vision transformer attention and HRNet in the encoding process. This integration successfully extracts global contextual information while maintaining high resolution spatial details leading to accurate segmentation of the kidney region.
- The contrast optimized PDN model used masked kidney images to segment the mass region. This model includes multiscale pooling, contrast enhancement, boundary refinement through separable convolutions and batch normalization along with feature fusion leading to segmentation of mass boundaries and greater structural localization.
- The proposed VHUCS-Net model implements a feature fusion method combining the mass segmentation output from the contrast optimized PDN with the kidney region segmented by the standard enhanced U-Net model. This fusion enhances boundary reliability, identifies structural differences and enables robust multi scale feature representation.
- The proposed VHUCS-Net model is evaluated using a kidney segmentation dataset which systematically evaluate the model efficacy through multiple features broad validation and comparison analysis are performed using publicly available datasets.

This research paper is structured as follows: Section 2 presents a focussed review of the existing kidney segmentation techniques with a comparative table including datasets, methods, imaging modalities, evaluation criteria while highlighting their limits and key contributions. Section 3 defines the proposed VHUCS-Net architecture and explains its internal components and structural design. Section 4 includes results and discussion that details the dataset analysed the training and validation methodologies, the evaluation criteria and the performance analysis. It includes validation using publicly available dataset an ablation study, comparisons with state-of-the-art models, and illustrate both visual and quantitative results. Section 5 concludes and highlights the future directions.

## 2 Related work

Kittipongdaja and Siriborvornratanakul (2022) performed a study using 2.5D ResU-Net and 2.5D DenseU-Net architectures attaining a dice score of 0.95 on the KiTS19 dataset and 0.87. Hatsutani (2023) proposed a framework with three networks such as a base network to generate initial tumor masks, a PDN for recognizing protruded areas and a fusion network for the final prediction of tumor masks. The proposed technique attained a dice score of 0.615 and a sensitivity of 0.721 on the KiTS19 dataset.

Bolocan et al. (2023) evaluated a U-Net architecture for tumor segmentation and attained a mean dice score of 0.675 representing moderate segmentation precision. The ResNet101 classifier had an accuracy of 88.5% in diagnosing. Swain et al. (2024) conducted a study on automated instance segmentation of glomeruli in renal images using YOLOv8 with Mask R-CNN. Both models underwent training and validation using the human vasculature dataset. Performance review shown that YOLOv8 outperformed Mask R-CNN attaining a precision of 0.97 over 0.85 a recall of 0.85 over 0.78 and a mean average precision at IoU 50 of 0.93 over 0.85.

Oghli et al. (2024) developed Fast U-Net++ which attain segmentation accuracy attaining dice coefficients of 0.97 for sagittal views and 0.95 for axial views therefore providing the prediction of kidney size and volume. Zhao et al. (2020) implemented a Multi-Scale Supervised U-Net (MSS U-Net) a 3D U-Net architecture designed for accurate tumor segmentation from CT scans. The model incorporates deep supervision with an exponential logarithmic loss function to improve training efficiency. During assessment using the KiTS19 dataset it attained a dice coefficient of 0.805 for tumor segmentation.

Zhao et al. (2023) proposed a cascaded architecture that integrates 3D U-Net which used to segment bilateral kidney borders and identify regions of interest and then an ensemble of 3D U-Nets was used to detect and segment renal masses. A ResNet model was applied to classify the segmented masses based on their size. This method shown high productivity attaining dice scores of 0.99 for kidney segmentation and classification accuracies of 86.05% for lesions under 5 mm and 91.97% for lesions 5 mm or greater. Conze et al. (2024) proposed a methodology that encompasses three categories of network architectures: CNN-based, transformer-based and hybrid CNN/transformer based models. The methodology used a dual-task learning framework, where a shared extractor paired with individual decoders enabled efficient processing. The models were evaluated using various MRI dataset, with Swin U-NetV2 exhibiting superior performance by obtaining a dice similarity score of 0.931.

Hsiao et al. (2022a) evaluated EfficientNet-B5 as the encoder and a feature pyramid network as the decoder, evaluated on the 3D-IRCADb-01 dataset. The model shows robust performance across all parameters attaining a dice score of 91.50, a recall of 96.43, an accuracy of 87.22% and an IoU score of 84.42. Hsiao et al. (2022b) implemented a modified U-Net architecture that incorporates ResNet-41 and EfficientNet as the encoder. The method employs statistical hounsfield unit windowing and image screening techniques to improve the preprocessing phase. Experimental attaining a dice score of 0.9648 for kidney segmentation and 0.7294 for tumor segmentation along with a minimal kidney volume error of 0.014.

Patel et al. (2024) proposed a framework using 3D-TR-DU-Net++ for kidney image segmentation and Adaptive and Attentive Residual DenseNet with Gated Recurrent Unit (AA-RD-GRU) for classification optimized through the (modified crayfish optimization algorithm. This method a dice score of 0.9470 for kidney segmentation and 0.6099 for tumor segmentation). Hussain et al. (2021) utilized a selection based convolutional neural network to analyze kidney vertical dimension, further using a hybrid sagittal-axial Mask R-CNN to generate a 3D bounding box of the organ. The method showing a kidney boundary localization error of 2.4 mm and a mean volume estimation error of 5%.

Jariwala et al. (2024) executed and trained U-Net and DeepLabv3 + architectures. The evaluation results showed that DeepLabv3 + outperformed U-Net, with dice scores of 0.94 and 0.82, IoU values of 0.182 and 0.160 and training and validation losses of 0.3928 and 0.4488, respectively. Uhm et al. (2022) developed DiagnosisGAN a deep learning framework integrates a generator, a discriminator, and a lesion segmentation network all trained simultaneously with various loss functions. An evaluation classification accuracy (*p* < 0.05) and attained a mean AUC (mAUC) of 0.829 signifying superior diagnostic efficacy compared to conventional techniques.

Causey et al. (2021) implemented an ensemble of U-Net models attained dice scores of 0.601 on the local test set and 0.6099 on the competition test set for tumor segmentation which resulted in a combined dice score of 0.7784. Türk et al. (2020) developed a hybrid V-Net model that improves the traditional V-Net design by incorporating both ET-Net and Fusion V-Net. This approach attained dice coefficients of 0.977 for kidney segmentation and 0.865 for tumor segmentation.

da Cruz et al. (2020) applied a technique that combines U-Net for segmentation and AlexNet for classification incorporating a false positive reduction phase to improve accuracy. This approach resulting in an average dice coefficient of 0.9633, a jaccard index of 0.9302, a sensitivity of 0.9742, a specificity of 0.9994 and an accuracy of 99.92%. Chen et al. (2024) proposed TransUNet modifying the U-Net architecture through the integration of self-attention mechanisms. It employs a transformer encoder for global context extraction and a decoder for enhanced segmentation with the capability of including both 2D and 3D formats. TransUNet attained average dice of 0.0106 and 0.0430.

Sharma et al. (2017) developed a CNN-based architecture attained a mean dice similarity value of 0.86 and a high correlation value of 0.98 for total kidney volume data thereby validating its accuracy and consistency. Mehedi et al. (2022) explored U-Net and SegNet designs for segmentation along with transfer learning model for classification. Among U-Net attained an accuracy of 97.58%, an IoU of 0.9857 and a dice score of 0.5440. In classification tasks, VGG16 exceeded the other models with an accuracy of 99.48%, a sensitivity of 0.9921, and a specificity of 0.9961. Zhang et al. (2020) introduced a two-stage coarse-to-fine methodology for kidney segmentation in CT images. Initially, whole CT slices were standardized to a uniform size for initial segmentation. During the second stage, the slices were resampled and cropped into smaller patches for the purpose of fine-grained segmentation. The model was trained on 168 CT scans and assessed using 42 test images, attaining an average dice similarity coefficient of 0.9453 indicating efficient segmentation ability.

Yang et al. (2025) proposed MUNet which achieved the highest dice similarity coefficient value of 0.915 and the highest Hausdorff95 value of 6.437 across the BraTS2020 and BraTS2018 datasets. Pimpalkar et al. (2025) built a fine-tuned deep learning framework integrating transfer learning models AlexNet, VGG16, InceptionV3 and ResNet50 attaining a highest accuracy of 99.96%. Vezakis et al. (2024) proposed a combination of 3D Attention U-Net and 2D U-Net for automated segmentation of organs in FDG-PET images achieving a dice score of up to 97% for brain and bladder segmentation. Shelke et al. (2025) proposed Ensemble EfficientNet combining multiple EfficientNet models through ensemble learning for diabetic retinopathy detection achieving an accuracy of 95% and a recall of 97%. Table 1 shows a comparison of deep learning kidney segmentation methods by technique, modality and performance.

**Table 1:** Comparative summary of kidney segmentation methods across various datasets and imaging modalities.

Ref	Dataset	Methodology	Imaging modality	Metrics	Key contribution
Kittipongdaja and Siriborvornratanakul (2022)	KiTS19, Thai Patient	2.5D ResU-Net and 2.5D DenseU-Net	CT	Dice Score: 0.95(KiTS19), 0.87 (Thai)	Combines spatial efficiency with contextual depth
Swain et al. (2024)	HuBMAP	YoLOV8 and Mask R-CNN	Histopathology	YOLOv8 Recall: 0.85, mAP50: 0.93; Mask R-CNN, Recall: 0.78, mAP50: 0.85	Uses mAP and IoU thresholds for precise localization.
Oghli et al. (2024)	Three Iranian imaging centers	Fast U-Net++	Ultrasound	Dice: 0.97 (sagittal), 0.95 (axial)	Segments kidneys and predicts five key length, width, thickness, volume, and parenchymal thickness measurements.
Zhao et al. (2020)	KiTS19	Multi-scale supervised 3D U-Net	CT	Dice: 0.805	Uses deep supervision with exponential log loss.
Zhao et al. (2023)	KiTS21	Cascading 3D U-Net and ResNet	CT	Renal mass Dice: 0.75–0.83, Recall: 0.84,	Improves accuracy through statistical analysis.
Conze et al. (2024)	Genkyst	CNN, Transformer, Hybrid with dual-task learning	MRI	Dice: 0.931	Shared encoder with per-kidney decoders.
Hsiao et al. (2022a)	KiTS19, 3D-IRCAD-01	EfficientNet-B5 encoder with FPN decoder	CT	Dice: 0.969	Lightweight model with optimized hyperparameters.
Hsiao et al. (2022b)	KiTS19	Modified U-Net with ResNet-41 and EfficientNet	CT	Kidney Dice: 0.9648, Tumor Dice: 0.7294, Kidney volume error: 0.014	Uses HU windowing and advanced preprocessing.
Patel et al. (2024)	KiTS21	3D-TR-DU-Net++ and AA-RD-GRU with MCOA	CT	Kidney Dice: 0.9470, Tumor Dice: 0.6099	Transformer attention for temporal dependencies.
Jariwala et al. (2024)	KiTS23	U-Net and DeepLabv3+	3D CT	DeepLabv3 + Dice: 0.94, IoU: 0.82; U-Net Dice: 0.82, IoU: 0.0182	ASPP refines boundaries of complex tumors.
Uhm et al. (2022)	The Cancer Imaging Archive (TCIA)	DiagnosisGAN (3D U-Net)	CT	Mean AUC (mAUC): 0.829	Initial feature maps improve lesion identification.
Causey et al. (2021)	KiTS19	Ensemble of U-Net models	CT	Kidney Dice: 0.9470, Tumor Dice: 0.6099	Combines U-Nets to boost consistency.
Türk et al. (2020)	KiTS19	Hybrid V-Net with fusion V-Net and ET-Net	CT	Kidney Dice: 0.977, Tumor Dice: 0.865	Fusion encoding with edge-aware decoding.
da Cruz et al. (2020)	Local dataset, KiTS19	AlexNet + U-Net	CT	Local dataset: Dice: 0.963, KiTS19: Dice: 0.930	Classifier reduces false positives.
Sharma et al. (2017)	ADPKD patient dataset	Automated deep learning segmentation	CT	Dice: 0.86,	Robust TKV quantification.
Zhang et al. (2020)	KiTS19	Coarse-to-fine segmentation with CNNs	CT	Dice: 0.945	Two-stage segmentation with correction.

### 2.1 Limitations of existing kidney segmentation approaches

The key challenges in kidney mass segmentation is performed using VHUCS-Net architecture for enhancing cross-modality robustness is given as below:

- A significant challenge lies in the generality of existing algorithms being trained and validated on similar kidney segmentation datasets and imaging modalities. The lack of diversity limits their generalizability reducing efficacy in real clinical environments where models must exhibit robustness across diverse datasets and varying imaging conditions.
- Kidney tumor segmentation undergoes difficulties due to irregular shapes, small lesion sizes, and unpredictable intensity patterns. These characteristics consistently interrupt accurate border identification resulting in minimized segmentation precision, lower model sensitivity and less dice coefficients mainly in the identification of insignificant tumor patches.
- Medical imaging modalities including PET, CT, ultrasound and MRI exhibit distinctive characteristics representing significant challenges to the development of a general segmentation model. The modality specific differences require suitable preprocessing and architectural change thereby increasing the density of model implementation and reducing multi-modality flexibility.
- One of the main challenges is the accurate identification of tiny masses that occur in the initial stages. These abnormalities result in decreased sensitivity and specificity or leading to false positives. As a result, clinical reliability has been reduced which may lead to delayed diagnosis or inaccurate treatment decision.

To address the key challenges in automatic kidney mass segmentation this research paper proposes the VHUCS-Net model that includes a transformer enhanced U-Net that combines the strengths of the standard U-Net with ViT and HRNet features. This integration enables the extraction of global contextual information while preserving fine spatial features thus refining the segmentation accuracy of irregularly shaped and small kidney mass regions. To address the limitations related to low contrast and inaccurate boundary detection the model includes a contrast optimized PDN. This model uses multiscale pooling, contrast enhancement and boundary refinement to attain accurate segmentation of mass boundaries. A dual-track fusion method is used to fuse kidney and mass feature maintaining structural stability thus improving robustness across various imaging modalities.

## 3 Proposed methodology

This section contains a detailed overview of the proposed architecture focusing on the sequential design with its key mechanisms including feature extraction, feature fusion and segmentation modules.

### 3.1 Architecture overview

**Figure 1:**
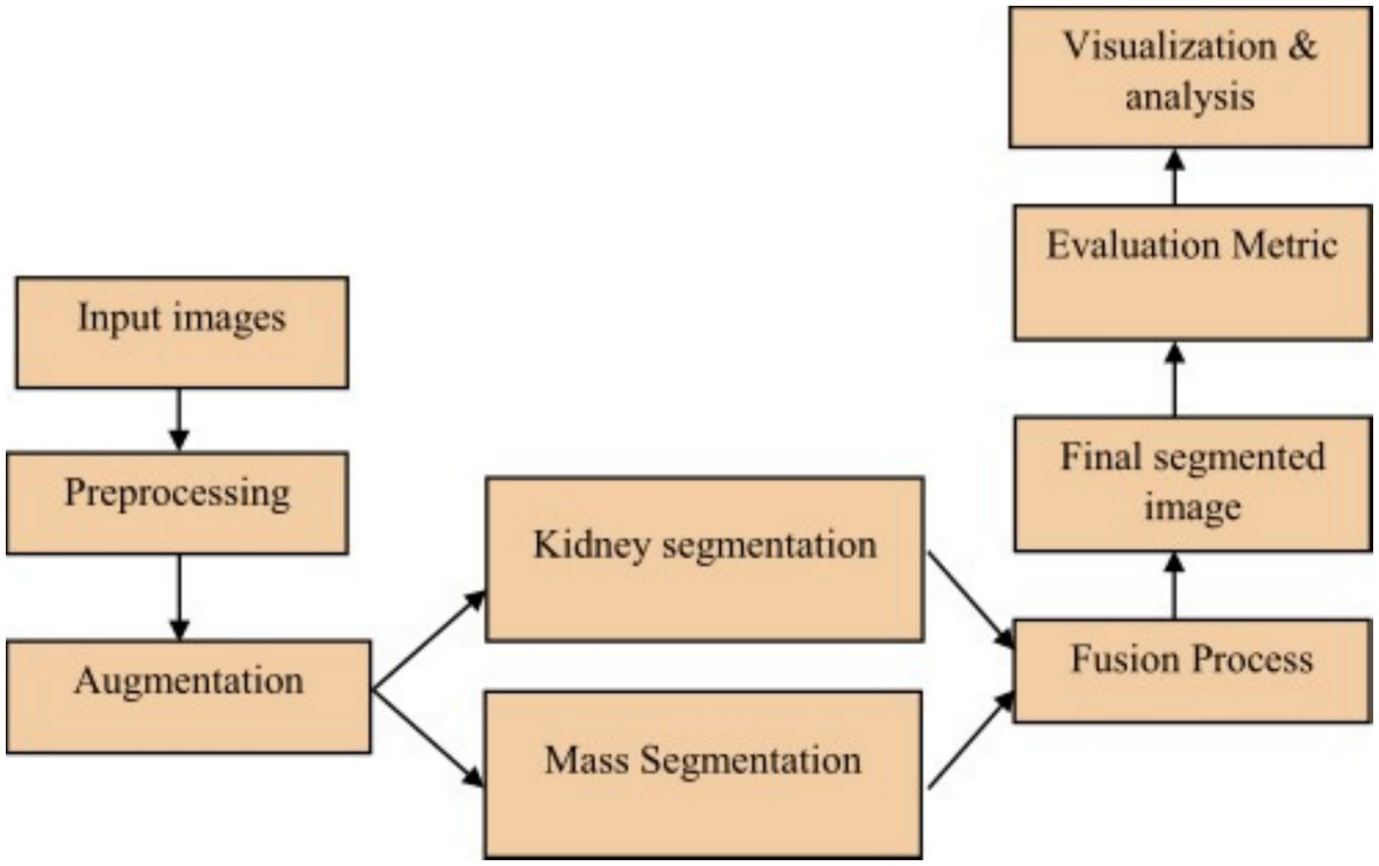
Workflow of proposed system.

**Figure 2:**
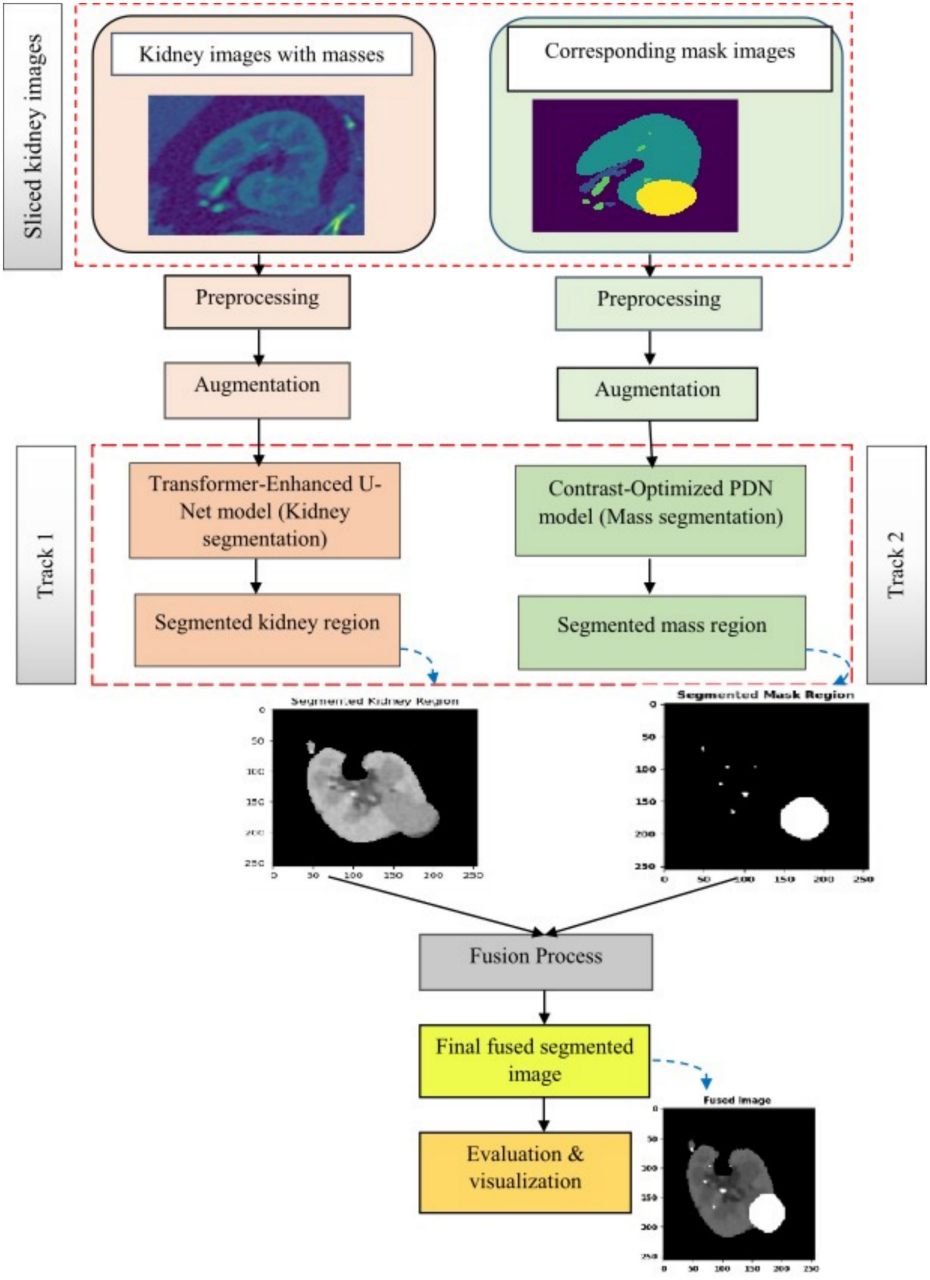
Overall architecture of the proposed model.

The proposed framework employs a dual-track architecture to improve kidney segmentation and mass localization. The sliced kidney images with the mass and their corresponding masks are preprocessed to minimize noise resulting in enhanced image quality which increases scalability for further analysis. Data augmentation is then applied on both inputs to reduce overfitting and enhance feature extraction resulting in improved model generalization as shown in Figure 1. The processed kidney image is input to track 1 which includes a transformer enhanced U-Net model that incorporates standard U-Net with ViT and HRNet layers in the encoder to attain accurate spatial reconstruction. The processed mask images are at the same time input into track 2 which uses a contrast optimized PDN model that integrates contrast enhancement and boundary refinement to accurately segment the specific mass region within the kidney. The outputs from both tracks are then fed into the fusion phase where the segmented kidney region from track 1 and the segmented mass region from track 2 are fused together to generate a refined and broad final segmentation. This integrated output provides a clearly defined kidney structure with the mass accurately segmented thereby enabling accurate detection and evaluation of the affected area. The combined result enhances overall diagnostic consistency as shown in Figure 2.

### 3.2 Preprocessing

Preprocessing is applied sequentially to both kidney image $I_{k}$ to enhance structural visibility and support feature learning. The sequence includes contrast limited adaptive histogram equalization (Buriboev et al., 2024) followed by global histogram equalization producing a contrast enhanced representation suitable for further processing shown in Equation 1.

$$I_{k}^{\left(p\right)}=H(C(I_{k}))$$ (1)

The corresponding mask $M_{k}$ does not undergo any contrast enhancement. To preserves its original spatial integrity, the mask is carried forward without applying CLAHE/HE and only resizing and normalization are performed during data preparation. This is shown in Equation 2.

$$M_{k}^{\left(p\right)}=(M_{k})$$ (2)

To avoid data leakage the PDN branch receives the masked kidney image obtained by multiplying the preprocessed kidney image with the predicted mask $\hat{M_{k}}$ . This is defined in Equation 3.

$$I_{k}^{\mathrm{PDN}}=I_{k}⊙\hat{M_{k}}$$ (3)

The PDN input mainly depends on the predicted mask produced by the transformer enhanced U-Net ensuring that no ground-truth mask will be shown during inference.

### 3.3 Augmentation

Augmentation is applied consistently to the preprocessed kidney image $I_{k}^{\left(p\right)}$ and its corresponding mask image $M_{k}^{\left(p\right)}$ to improve the model generalization and robustness. The augmentation operation transforms these input as define in Equations 4, 5.

$$I_{k}^{\left(a\right)}=A\;(I_{k}^{\left(p\right)})$$ (4)

$$M_{k}^{\left(a\right)}=A\;(M_{k}^{\left(p\right)})$$ (5)

Here, A(⋅) denotes the augmentation operator which includes a series of spatial and intensity transformation. Horizontal and vertical flips introduce positional variation enabling the model to learn invariant features based on the patient positioning and scan orientation. Rotational augmentation within a $\pm 20^{{}^{\circ}}$ range adjusts alignment inconsistencies and enhances robustness to angular variations. Random modifications in brightness and contrast replicate various lighting conditions enhancing the model flexibility to changing image intensities. Also, elastic transformations result in complex non-linear changes while maintaining anatomical integrity thus enhancing feature diversity and generalization ability.

### 3.4 Segmentation workflow

The proposed hybrid VHUCS-Net architecture features two parallel processing tracks: a transformer enhanced U-Net model and a contrast optimized PDN model. The transformer enhanced U-Net combines the standard U-Net architecture with a vision transformer layer for global context acquisition and HRNet to maintain spatial resolution and complex details. The contrast optimized PDN model integrates multi scale max pooling, contrast enhancement and boundary refinement to increase localized mass segmentation. The dataset consists of two types of inputs: kidney images with masses and the corresponding mask images. Both input types are given preprocessing and augmentation to enhance data quality and augment model robustness. The processed kidney images are input into the transformer enhanced U-Net model while the processed mask images are given to the contrast optimized PDN model. This dual-track technique ensures corresponding feature extraction and precise segmentation by using the features of both models thus improving overall efficacy in kidney mass segmentation.

#### 3.4.1 Transformer enhanced U-Net model

The transformer enhanced U-Net model which segments the kidney region by integrating standard U-Net with ViT and HRNet enabling the parallel extraction of global sematic information and detail structural features. The input to this model is the augmented kidney image $I_{k}^{\left(a\right)}$ a preprocessed and augmented image with spatial dimensions of 224 × 224 × 3 normalized to [0,1] representing height and width as shown in Equation 6. The processed input is subsequently passed through the model layers to perform accurate kidney region segmentation.

$$I_{k}^{\left(a\right)}\in R^{224\times 224\times 3}$$ (6)

##### 3.4.1.1 Encoder

The encoder analyses the input image $I_{k}^{\left(a\right)}$ using a hierarchical framework which integrates ViT attention mechanisms with HRNet-based convolutions to extract high-resolution features at each encoding level. The input image $\mathrm{X\epsilon}R^{224\times 224\times 3}$ is first transformed into an initial feature map $F_{0}$ using an embedding layer as shown in Equation 7 as patch embedding layer. Here, $F_{0}$ serves as the starting point for the first encoder block. For subsequently encoder blocks i the input is the output from the previous block denoted $F_{i-1}$ .

$$F_{0}=\text{Embed}(I_{k}^{\left(a\right)})$$ (7)

$$\begin{array}{l} F_{0}=\text{Embed}(I_{k}^{\left(a\right)})=\\ \text{Conv}2D(\text{filters}=C,,{\text{Kernel}}_{\text{size}}=P,,\text{stride}=P)(I_{k}^{\left(a\right)})\;\; \end{array}$$ (8)

**Figure 3:**
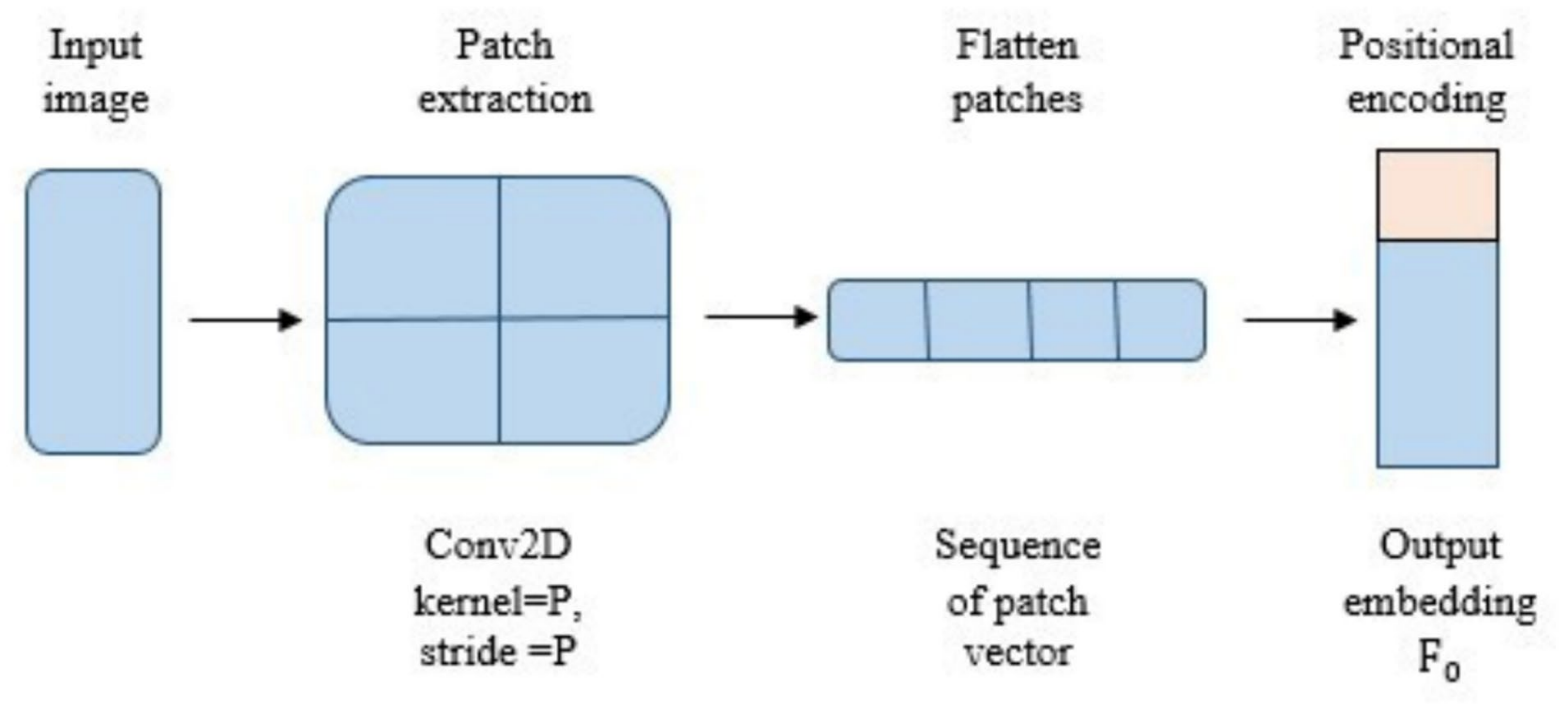
Schematic representation of the embedding layer.

In Equation 8, P × P denotes the patch size and C represents the embedding dimension and the stride is equivalent to the patch size to ensure non overlapping patches. The embedding layer divides the input image into non overlapping patches through a conv2D layer and maps each patch to a feature vector. Positional encoding is incorporated to preserve spatial information resulting in the patch embedding $F_{0}$ for the encoder as illustrated in Figure 3.

**Figure 4:**
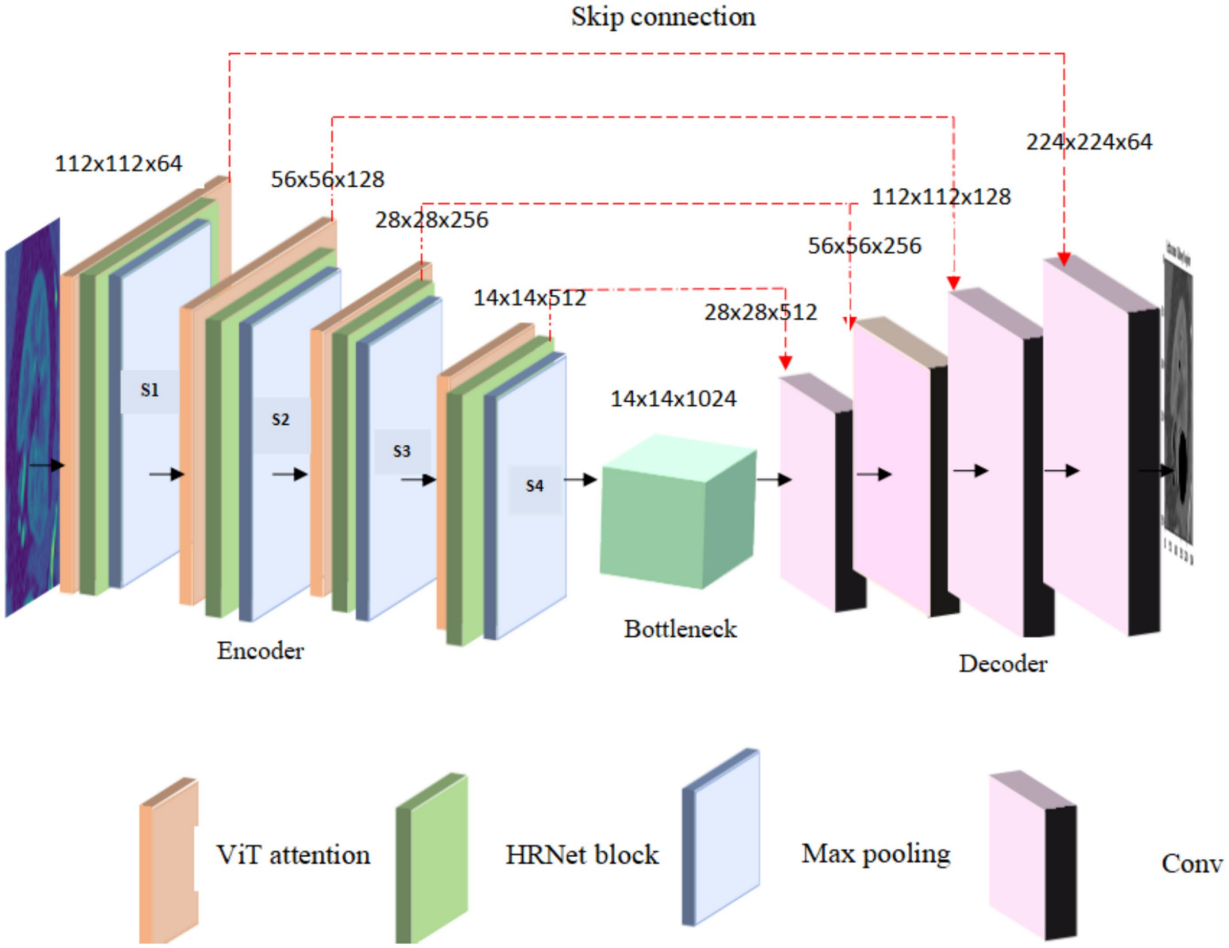
Transformer enhanced U-Net model frame work.

Max pooling is used at each level to reduce spatial dimensions while maintaining essential details enabling the model to effectively capture global contextual information and local structural variations. The ViT attention mechanism captures long range dependencies and the resulting HRNet layer maintains fine grained spatial details. The max pooling reduce the spatial dimensions to 112 × 112 and increase in feature depth 64. This is followed by an additional sequence of ViT attention and HRNet processing which further increases representations. The spatial resolution is reduced to 56 × 56 and by an increase in feature depth to 128 enhancing the model capacity to capture local texture. As the encoding progress the resolution decreases to 28 × 28 and then to 14 × 14 while the feature depth increases to 256 and 512, respectively, as shown in Figure 4.

**Figure 5:**
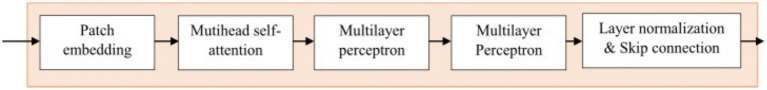
Internal working process of the ViT attention block.

The ViT which captures global dependencies from the input feature map $F_{i-1}$ . This process is illustrated in Figure 5 is performed using multi head self-attention and feed forward network where the input undergoes layer normalization is then processed by multi head self-attention as expressed in Equation 9. The resulting output is then refined through the feed forward network while maintain a residual connection as explained in Equation 10.

$$\mathrm{ViT}\;(F_{i-1})=\text{MHSA}\;(\mathrm{LN}(F_{i-1}))+F_{i-1}$$ (9)

$$F_{i}^{\mathrm{ViT}}=\mathrm{FFN}\;(\mathrm{LN}\;(\mathrm{ViT}\;(F_{i-1})))+\mathrm{ViT}\;(F_{i-1})$$ (10)

**Figure 6:**
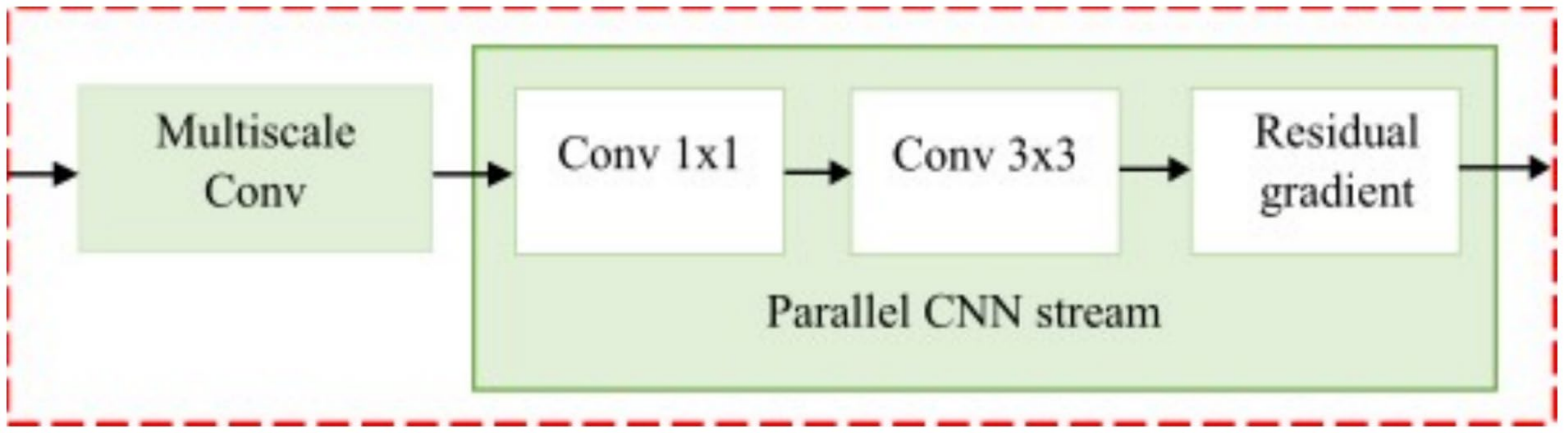
Layer composition of the HRNet block.

Following ViT attention HRNet subsequently refines the extracted features using multi scale convolution as illustrated in Figure 6. Let S denote the number of scales. Multiple convolutional scale filters $W_{s}$ operate at different resolutions to enhance feature representation as expressed in Equation 11.

$$F_{i}^{\mathrm{HR}}=\sum_{s=1}^{s}W_{s}*F_{i}^{\mathrm{ViT}}$$ (11)

The first encoder stage produces a feature map of 112x112x64 with the high resolution level. As the encoding progress, the spatial resolution is progressively reduced while the feature depth increases by $R^{\left(112\times 112\times 64\right)}$ , $R^{\left(56\times 56\times 128\right)}$ , $R^{\left(28\times 28\times 256\right)}$ , $R^{\left(14\times 14\times 512\right)}$ sequential levels as shown in equation 12. This hierarchical transformation enables the network to capture of both comprehensive context and complex details. Such progressive encoding facilitates the integration of global context with local anatomical information enhancing the model precision in kidney mass segmentation.

$$F_{i}\in R^{H_{i}\times W_{i}\times C_{i}}$$ (12)

##### 3.4.1.2 Bridge

The bottleneck serves as an intermediary stage between the encoder and decoder performing feature compression and transformation. The major function is to reduce feature dimensionality while retaining essential information which allows efficient processing before the expanding of feature maps in the decoder. The bottleneck encodes high dimensional information into a compact representation ensuring that only the most essential and distinct characteristics are transmitted for decoding.

$$B_{i}=\sigma (w_{b}*F_{4}+b_{b})$$ (13)

In Equation 13, $B_{i}\in R^{14\times 14\;\;\times 1024}$ represents the corresponding bottleneck feature map $w_{b}$ and $b_{b}$ represent the convolutional weights and biases, respectively, and ∗ indicates the convolution process. The function σ corresponds to the ReLU activation function. This method allows the bottleneck to function as an intermediate point between feature extraction in the encoder and the reconstruction process in the decoder enabling optimal transfer of essential feature representations.

##### 3.4.1.3 Decoder

In the decoder phase, the transformer enhanced U-Net model progressively reconstruct the segmentation map through stepwise upsampling and element wise feature addition. At each stage (iϵ{4,3,2,1}) the feature map from the previous decoder layer $B_{i+1}$ is upsampled using transposed convolutions. To preserve fine-grained spatial details with their feature map $F_{i}$ is then added to the upsampled decoder feature map at the same resolution. This skip connection provides efficient feature fusion by reducing parameter and modifying redundancy while holding essential structural information as shown in Equation 14. The final segmented kidney region denoted as $K_{\mathrm{seg}}\in R^{224\times 224\times 64}$

$$S_{k}=\sigma [W_{d}^{T}(\text{upsample}\;(B_{i+1})+F_{i})+b_{d}]$$ (14)

where $B_{i+1}\;$ represents the decoder feature map from the stage $W_{d}^{T}$ is the transpose of the decoder weight matrix, $b_{d}$ is a learnable bias term and σ(⋅) denotes the activation function. This fusion process enhances spatial consistency maintains a balanced representation of global and local features and improves segmentation accuracy. Table 2 highlights the key differences between the original U-Net and the proposed transformer enhanced U-Net.

**Table 2:** Comparison between U-Net and transformer enhanced U-Net.

Component	Original U-net	Proposed transformer enhanced U-net
Overall architecture	Symmetric U-shaped encoder-decoder CNN	U-shaped encoder-decoder augmented with vision transformer and HRNet blocks
Input	Image of size H × W × C	Preprocessed image of size 224 × 224 × 3, split into patch embeddings and augmented
Encoder	Convolution + ReLU + Max pooling	Patch embedding → ViT attention → HRNet multi-scale convolutions → progressive downsampling
Feature extraction	Local features through convolution	Both global (ViT) and local (HRNet) features, hierarchical encoding with increasing depth (64 → 512)
Bottleneck	Convolutional layers at lowest resolution	Convolution + ReLU compressing encoder features while retaining essential
Decoder	Transposed convolution + skip connections	Transposed convolution + additive skip connections fusing encoder features efficiently
Skip connections	Concatenate encoder features	Additive fusion to preserve fine-grained details and reduce redundancy
Attention mechanism	None	Multi-head self-attention in ViT blocks for capturing long-range dependencies
Spatial detail preservation	May lose details due to pooling	HRNet maintains high-resolution features at multiple scales
Output	Segmentation map of original image size	Segmentation map of 224 × 224 × 64 with improved spatial and semantic accuracy

#### 3.4.2 Contrast optimized PDN model

**Figure 7:**
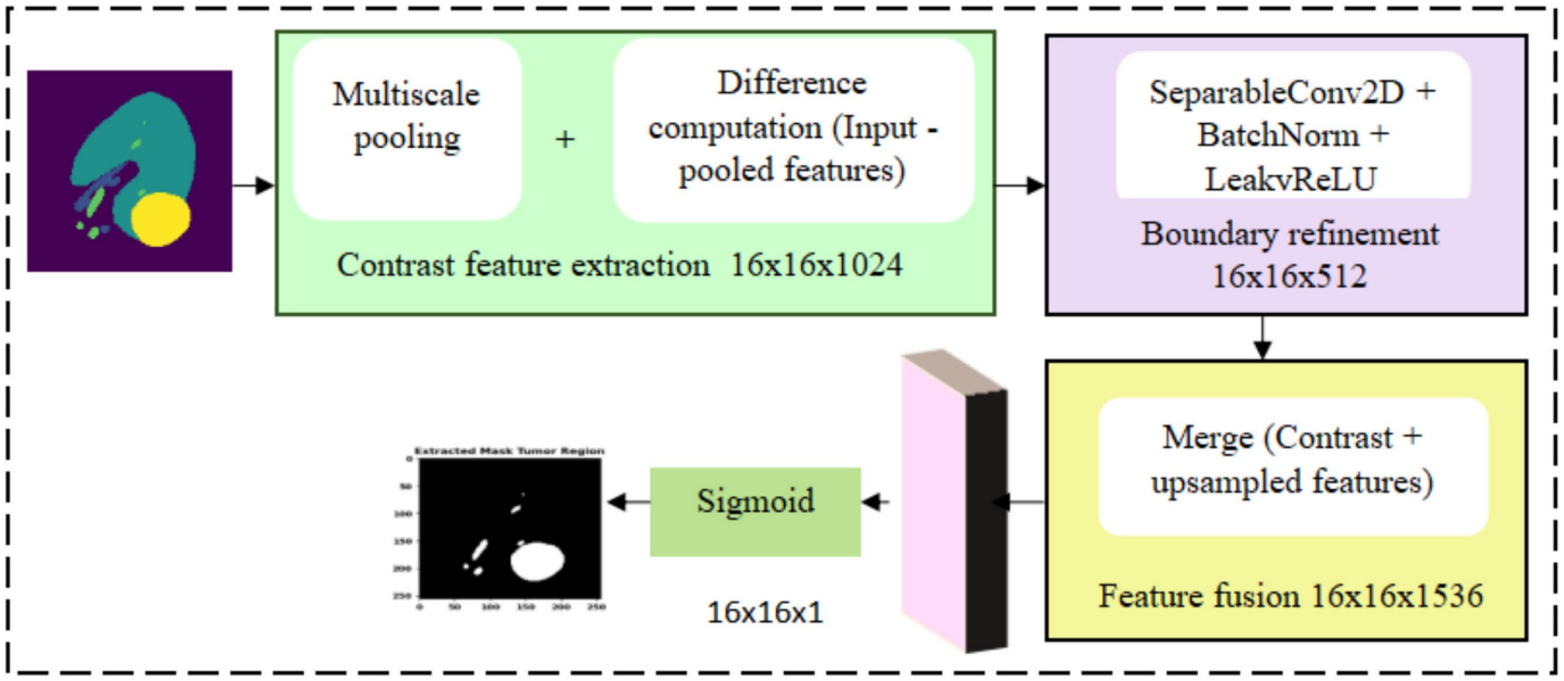
Contrast optimized PDN model design.

The contrast optimized PDN model segments the mass region from the augmented kidney mask image $M_{k}^{\left(a\right)}$ aiming to accurately detect and localize abnormal protrusions especially the kidney mass as shown in Figure 7. The process begins with feature extraction by applying a transformation function Θ to $M_{k}^{\left(a\right)}$ incorporating a normalization layer, activation function and convolutional filters. This operation is given by Equation 15 where W represents learnable convolutional filters, ∗ indicate convolutional operation, the bias term is denoted by b and f indicates the ReLU activation function. The resulting feature map $F_{\text{in}}$ is then processed by max pooling for attaining enhanced features.

$$F_{\text{in}}=\Theta (M_{k}^{\left(a\right)})=f(W*M_{k}^{\left(a\right)}+b)$$ (15)

**Figure 8:**
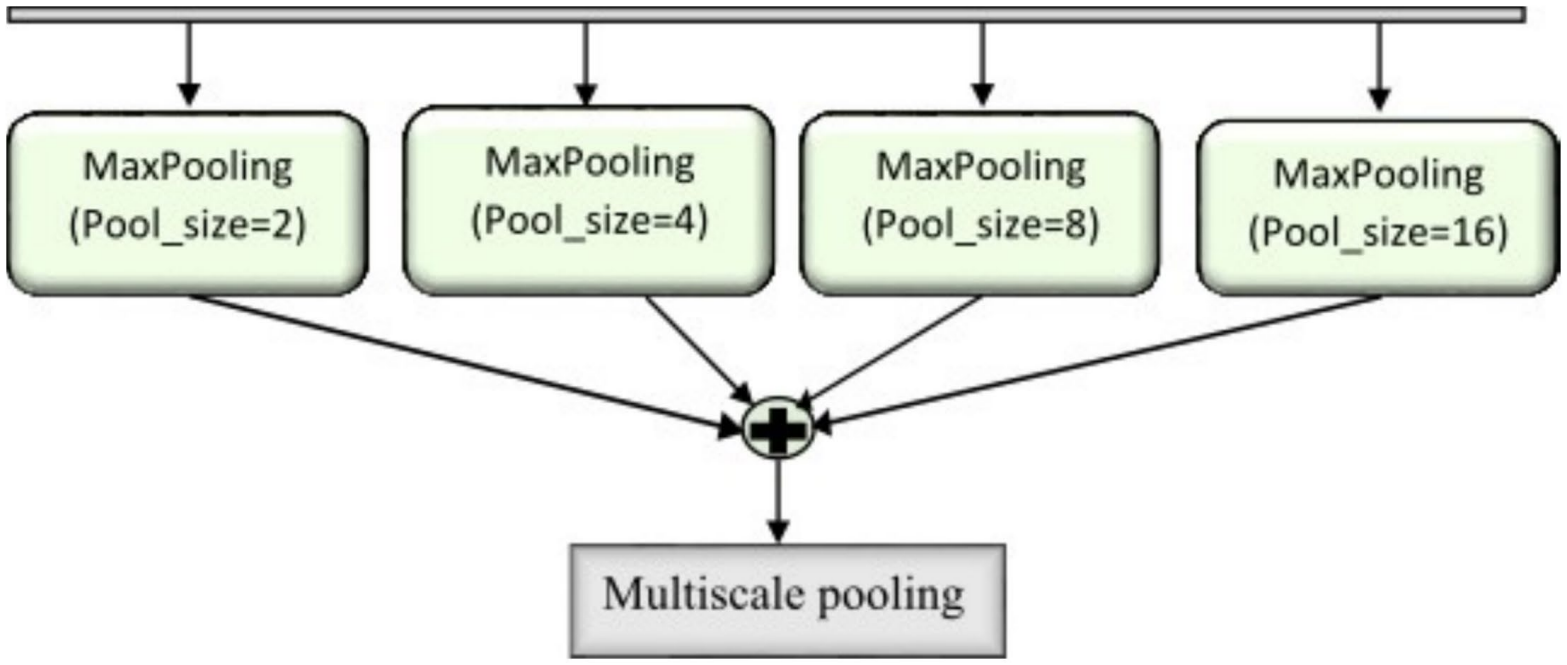
Representation of multiscale pooling mechanism.

Multiscale max pooling is applied to $F_{\text{in}}$ to capture features at different resolutions as shown in Figure 8 and the contrast between neighbouring regions is enhanced and refined through normalization, boundary refinement, and separable convolution for effective edge detection. The contrast feature extraction produces a feature map size 16x16x1024, capturing multiscale contrast information. The complete operation can be expressed as shown in Equation 16 where △ represent the difference operator. The resulting border refined feature map $F_{b}$ is activated using LeakyReLU.

$$F_{b}=\text{LeakyReLU}(\mathrm{BN}(\mathrm{SC}(\Delta (\mathrm{MP}(F_{\text{in}}))\;)))\;$$ (16)

A fusion operation combines the refined border features and upsampled contrast features to integrate high-resolution spatial details with enhanced contrast. This can be formulated as shown in Equation 17 where Φ represents a flexible fusion function. After concatenation, the feature fusion stage produces a 16x16x1536 integrating information from both branch. A finally a 1 × 1 convolution followed by a sigmoid activation produces the initial segmentation mask S and a thresholding step generates the final mass segmentation output $M_{\mathrm{seg}}$ where σ(⋅) is the sigmoid function and τ(⋅) denotes a thresholding operator obtained segmentation mask. The resullting attention mask has spatial dimension 16x16x1.

$$S_{m}=\tau [\sigma ({\text{Conv}}_{1\times 1}(\Phi (F_{b},\text{Upsample}(F_{b}))))]$$ (17)

The final segmentation mask integrates the kidney region from the transformer enhanced U-Net $S_{k}$ and the mass region from the contrast optimized PDN $S_{m}$ . A fusion operator Φ combine these output to ensure precise localization of masses within the kidney. The fused mask is refined to improve boundaries and correct misclassification pixels. The complete operation is expressed as shown in Equation 18.

$$S_{\text{final}}=\Phi (S_{k},S_{m})$$ (18)

## 4 Results and discussions

This section describes the experimental configuration specifying the dataset used for model implementation and the hyper parameters used during training. It further provides an ablation research to evaluate the impact on individual segmentation layers including the transformer enhanced U-Net and the contrast optimized PDN models. The evaluation metrics and analytical processes have been explained to effectively evaluate the performance of the proposed framework.

### 4.1 Experimental setup and system configuration

Experiments were performed in a notebook-based environment using an NVIDIA Tesla P100 GPU (16 GB VRAM), using CUDA 12.8 with fp32 precision. The batch size was set at 32, and the input resolution was maintained at 256 × 256 × 1 for all datasets. Under this configuration, the model required 0.3537 s per batch, resulting in an effective per-slice inference time of 0.0111 s (0.3537 s / 32). Given a 3D volume consisting of 30 consecutive 2D slices, the inference time per volume is 0.3316 s. Runtime was consistently evaluated at both the slice and volume levels, with the 16 GB VRAM.

### 4.2 Dataset description

**Figure 9:**
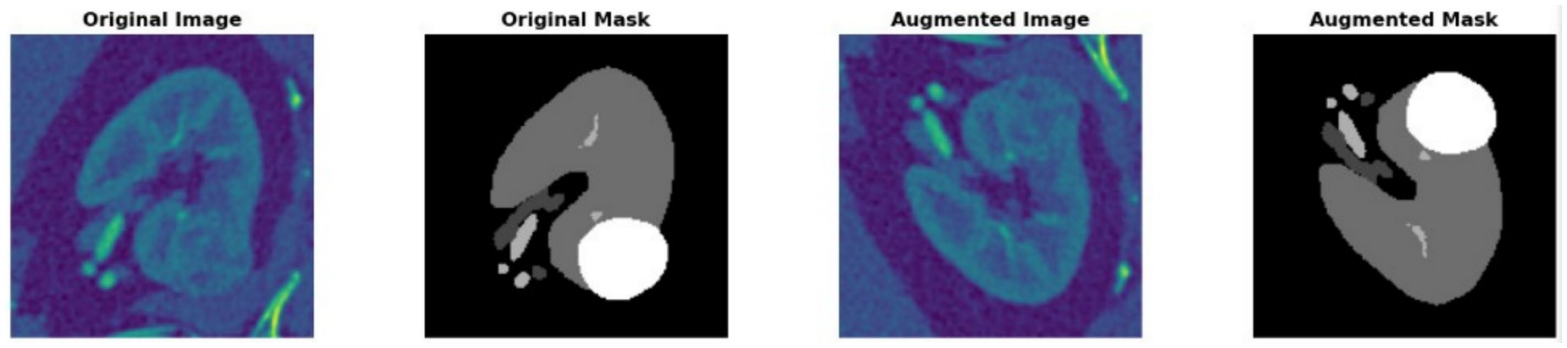
Original and augmented kidney images with the corresponding mask.

The dataset used in this study was obtained from a publicly available kidney segmentation dataset (Jadhav, 2023). It consists of two categories: sliced kidney images with masses and corresponding mask images as shown in Figure 9. The dataset included 4,054 images comprising 2,027 kidney images and their 2,027 corresponding mask images which contain tumor regions with no cases of tumor absence is detected. The sliced kidney images have an original resolution of 256 × 256 pixels with an average file size of approximately 20 KB. During preprocessing all images were uniformly resized to 224 × 224 × 3 before being fed into the model. The batch size was set to 32 for all experiments. The model contains 32.6 M parameters and the total computational cost is approximately 27.4 GFLOPs per forward pass. During the evaluation of the test dataset, the per-slice inference time ranged from 30 ms to 49 ms, resulting from several single-slice predictions.

### 4.3 Visual impact of preprocessing and augmentation

**Figure 10:**
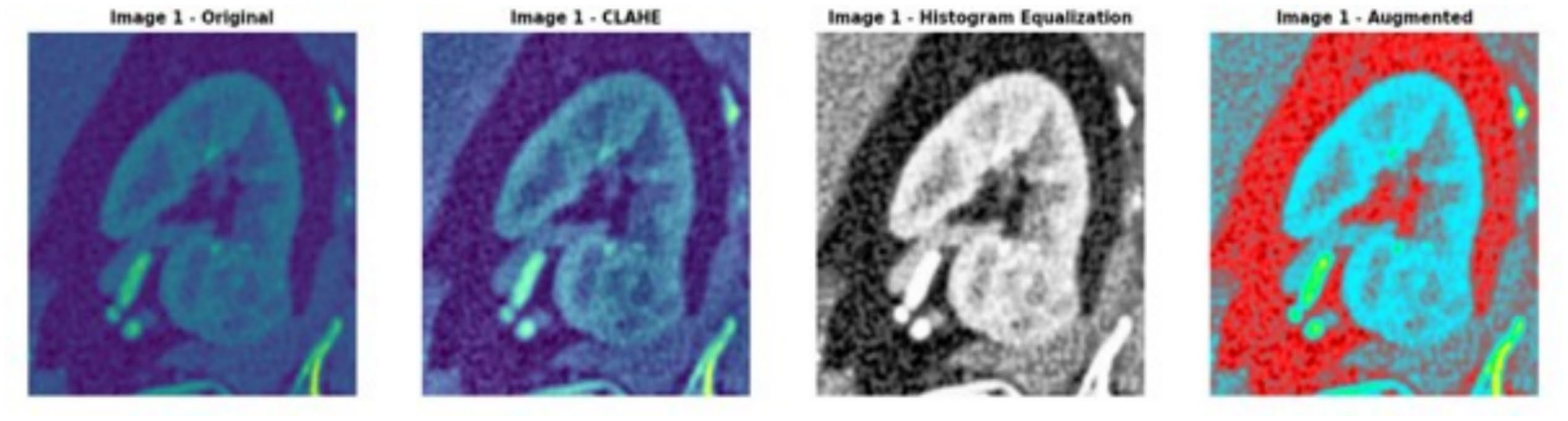
Image enhancement and augmentation on sliced kidney with mass.

The use of preprocessing and augmentation approaches was used to enhance the quality and diversity of the input data. The augmentation process included horizontal and vertical flip each applied with a probability of 50%, random rotations within ±20°, brightness and contrast modifications (30% probability) and elastic adjustments as shown in Figure 10. These augmentation methods together improve anatomical variation, intensity diversity and spatial alteration in the dataset. This technique improves stability and reduces the risk of overfitting by modeling changes in patient positioning, scanner parameters, noise and tissue contrast. The model starts to learn stable structural inputs based on static spatial or intensity patterns hence improving its ability to generalize to earlier identified cases. This method eventually enhances feature selection, robustness and overall prediction accuracy. Furthermore, contrast limited adaptive histogram equalization (Moradi et al., 2022) was applied with a 50% probability to augment local contrast hence enhancing model stability and optimizing feature extraction efficacy as shown in Table 3.

**Table 3:** Data augmentation and preprocessing impact.

Category	Parameter	Effect on dataset	Impact on memory	Computational complexity
Horizontal flip	*p* = 0.5	Doubles dataset with horizontal variations	Slight increase per batch	Minimal; very fast
Vertical flip	*p* = 0.5	Doubles dataset with vertical variants	Slight increase per batch	Minimal; very fast
Rotation	limit = ±20°, *p* = 0.5	Adds rotated variants; increases dataset by ~1.5–2×	Minor increase	Fast; small per-image cost
Random Brightness/contrast	*p* = 0.3	Increases diversity in intensity variations	Negligible	Low; minor pixel-wise operations
Elastic transform	alpha = 1, sigma = 50, alpha_affine = 50, *p* = 0.3	Adds geometric distortions; improves shape robustness	Moderate	Moderate; heavier than flip/rotation
CLAHE/Hist. equalization	clip_limit = 2.0, tile_grid = (8,8), *p* = 0.5	Enhances contrast; improves boundary visibility	Slight increase	Moderate; more intensive pixel processing
Rescaling	1./255	Normalizes intensity across all datasets	None	None
Learning rate	LR = 0.001	Stable convergence across all datasets	None	None
Batch size	32	Balanced training speed and memory usage	Moderate	Moderate
Epochs	35	Ensures consistent training duration	No additional impact per epoch	Standard training cost
Optimizer	Adam	Smooth gradient updates; avoids dataset-specific tuning	None	Low
Loss function	Dice loss	Improves segmentation consistency across modalities	None	Low
Metrics	IoU, dice coefficient	Uniform evaluation for all datasets	None	None
Mixed precision	float32	Ensures compatibility and numerical stability	None	None

**Figure 11:**
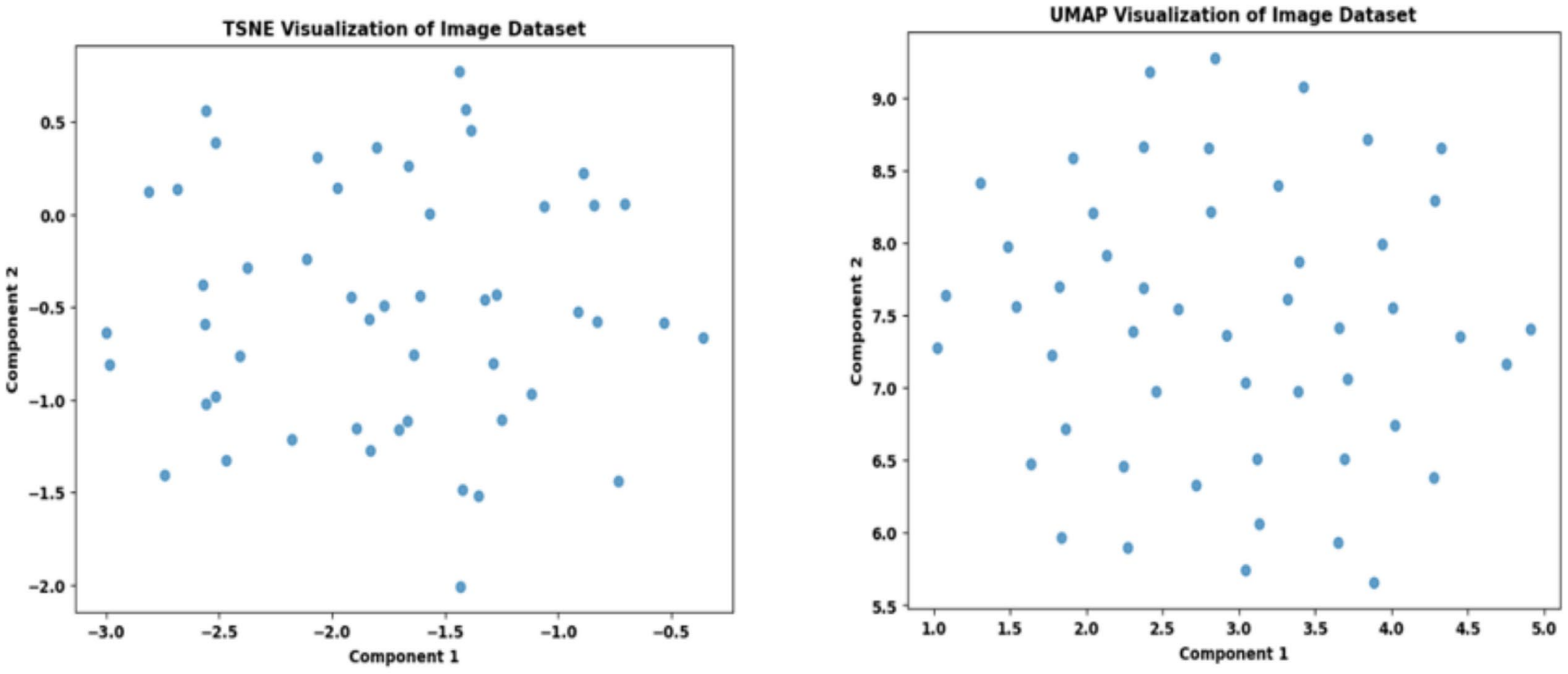
T-SNE and UMAP visualizations of kidney image.

To evaluate the efficacy of the preprocessing stage dimensionality reduction methods including t-SNE and UMAP were used for visualization. These approaches reduce the high-dimensional feature space into a two-dimensional space enabling an efficient visual evaluation of feature distribution and class partitioning. Figure 11 illustrates that processed kidney image provides well defined and significantly differentiated clusters with samples from identical classes closely packed and those from dissimilar classes widely spread. The refined cluster formation indicates superior feature quality therefore augmenting the model ability to differentiate normal kidney structures from malignant tumors. Table 4 displays five sample slices each illustrating the original images, mask image and predicted mask with overlaid red outlines thereby validating perfect segmentation.

**Table 4:** Sample images with mask and predicted contour overlay.

Visualization (original, mask, and predicted overlay)
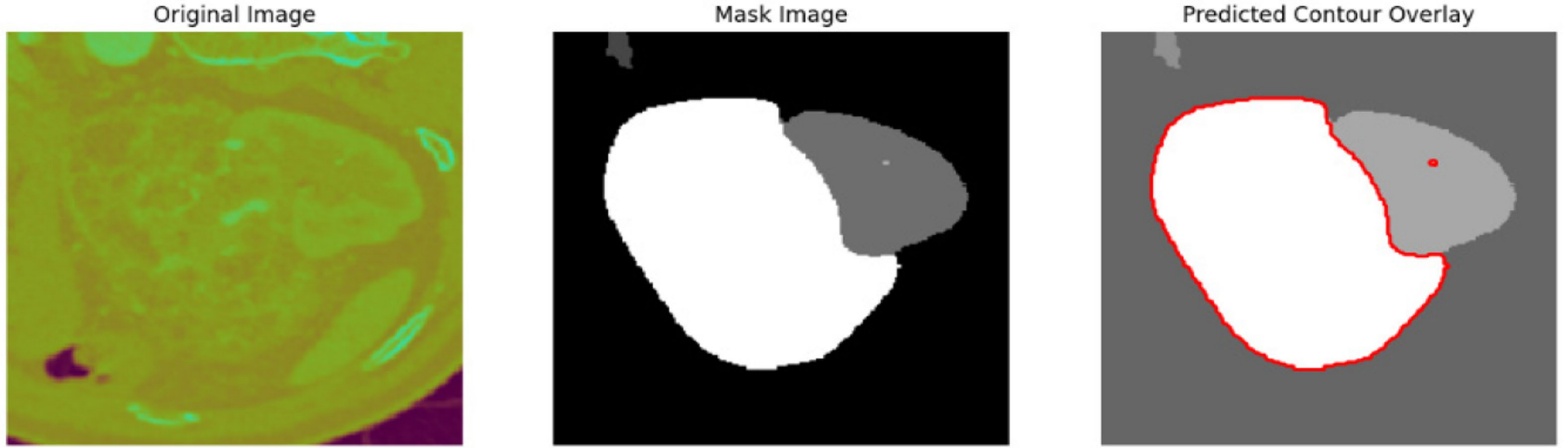
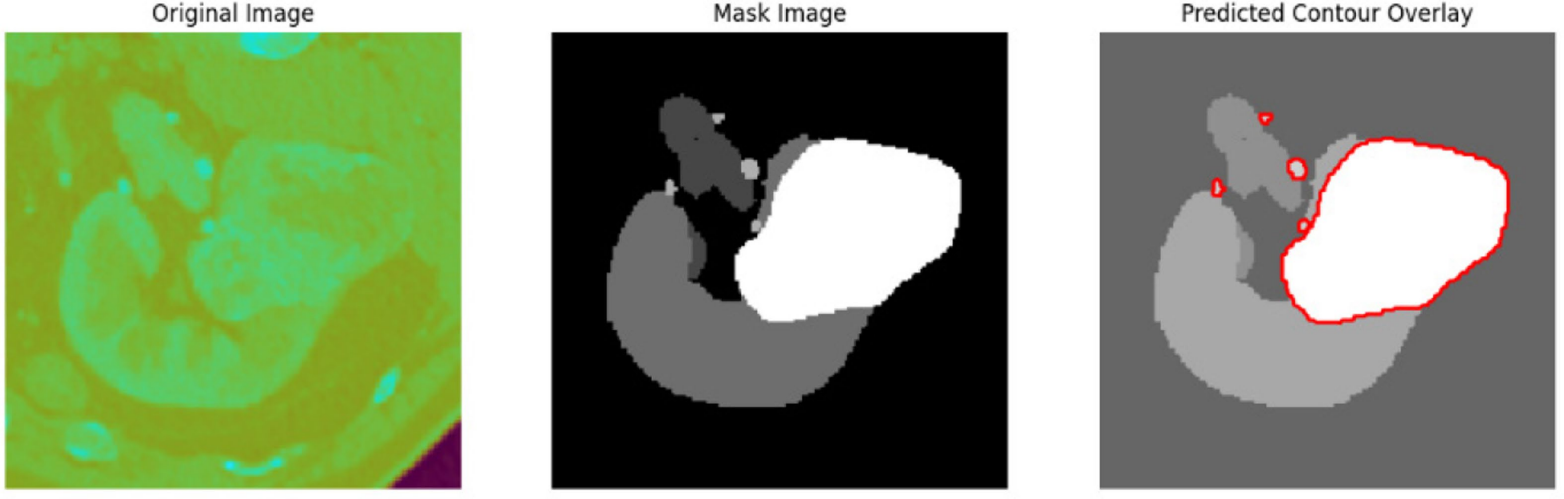
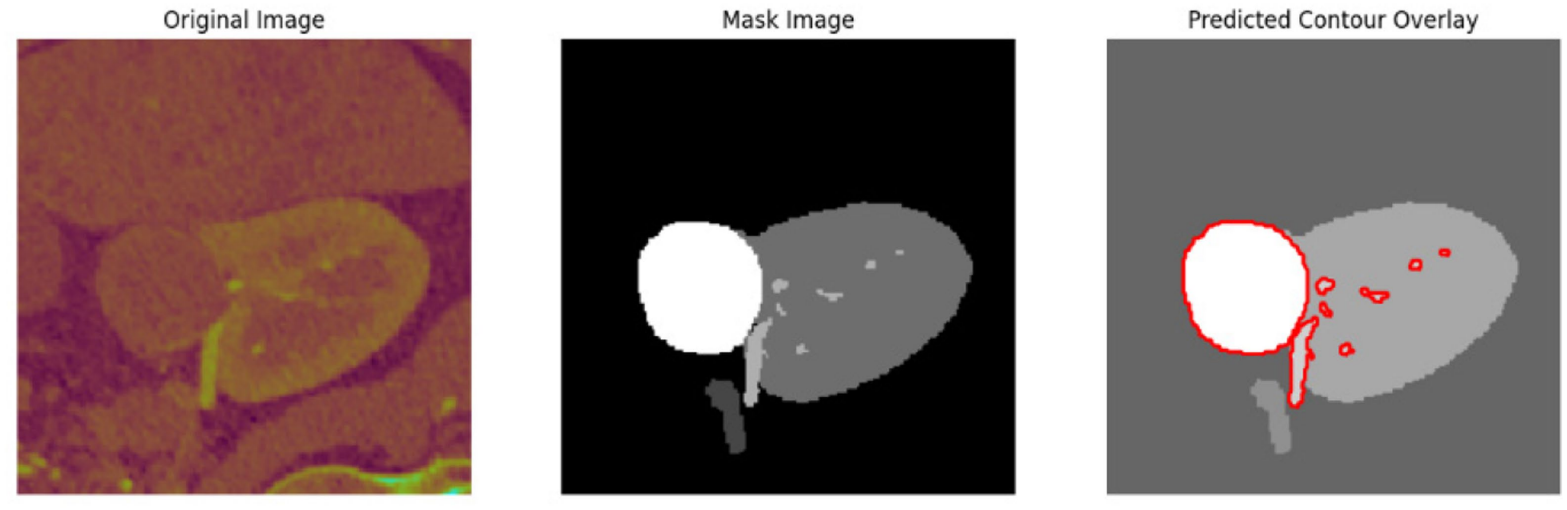
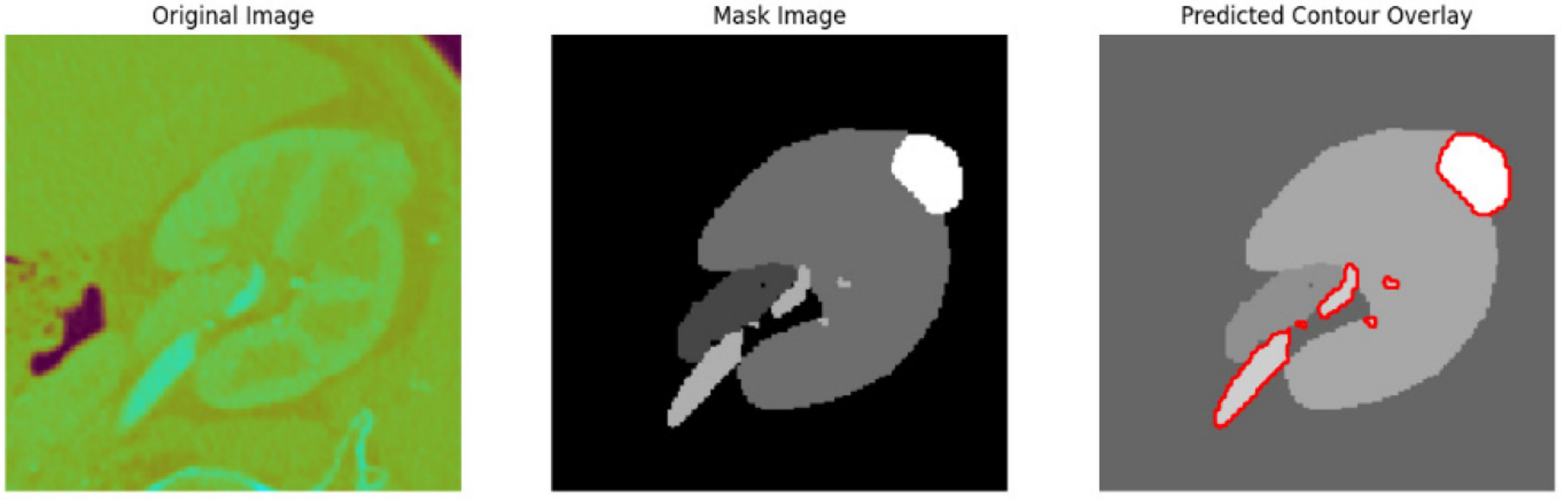
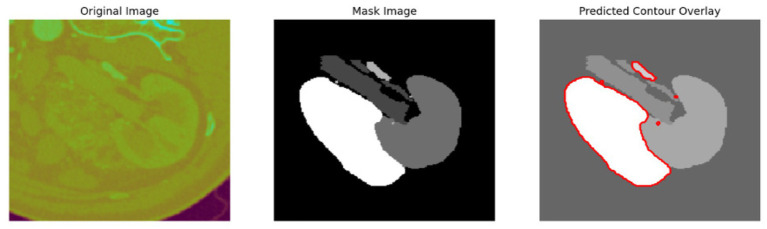

### 4.4 Hyper parameter tuning

In the segmentation phase the transformer enhanced U-Net was used for kidney segmentation whereas the contrast optimized PDN focused on tumor region segmentation. The fused model combines the outputs of both segmentation networks to improve IoU and dice coefficient scores while reducing loss (Müller et al., 2023). All three models (transformer enhanced U-Net, contrast optimized PDN, and the fused architecture) underwent training for 35 epochs with iterative weight adjustments throughout the dataset. A batch size of 32 was chosen to optimize computing efficiency and image resolution while the learning rate was fixed at 0.0001 (Tran et al., 2025) to ensure stable and consistent parameter convergence during training. Table 5 summarizes the network architecture, training hyper parameters and data preprocessing settings used for both ConD-PDN and VHU-Net models. It specifies layer configurations, activation functions, optimizer details, loss function, evaluation metrics, batch size, number of epochs and data augmentation strategies.

**Table 5:** Hyper parameter and configuration settings.

Layer	Hyper parameter	Value
Input layer	Input shape	(256, 256, 1)
Encoder—ConD-PDN	Filters (stage 1 → 4)	64 → 128 → 256 → 512
Encoder—VHU-Net	Filters (stage 1 → 4)	16 → 32 → 64 → 128
Conv2D (bridge)	Filters	1,024 (ConD-PDN), 128 (VHU-Net)
Criss-Cross attention	Activation	Sigmoid
Decoder—ConD-PDN	Filters (stage 1 → 4)	512 → 256 → 128 → 64
Decoder—VHU-Net	Filters (stage 1 → 4)	64 → 32 → 16 → 8
Output layer	Filters / Units	1
Output layer	Activation	Sigmoid
Optimizer	Type	Adam
Learning rate	LR	0.001
Loss function	Type	Dice loss
Metrics	Types	IoU, dice coefficient
Batch size	-	32
Epochs	-	35
Data augmentation	Rescale	1/255
Data augmentation	Rotation range	15°
Mixed precision	Policy	float32

### 4.5 Model training and validation

The training and validation processes of the proposed hybrid VHUCS-Net model were executed with uniform hyper parameter configurations. The framework first segments the kidney region from sliced images with masses using the transformer enhanced U-Net then segmenting the kidney mass from the masked images through the contrast optimized PDN model. The training set, representing 80% of the dataset is utilized to optimize model parameters while the validation set including 10% evaluates model performance during training and provides hyper parameter modification to prevent overfitting (Pavarut et al., 2023). The remaining 10% comprises the test set (Zhang et al., 2020) assigned for the final evaluation to measure the model generalization. Let N denote the total samples in the dataset while $T_{\text{train}},T_{\mathrm{val}},T_{\text{test}}$ denote the size of the training, validation and the test, respectively. The proportions for validation and splits are represent by $r_{\mathrm{val}},r_{\text{test}}$ ensure a balanced allocation for model training fine tuning and evaluation (Nagarajan and Ramprasath, 2024). The dataset splits are calculated as follows in Equations 19–21.

$$T_{\text{train}}=N\times (1-r_{\mathrm{val}}-r_{\text{test}})$$ (19)

$$T_{\mathrm{val}}=N\times r_{\mathrm{val}}$$ (20)

$$T_{\text{test}}=N\times r_{\text{test}}$$ (21)

### 4.6 Evaluation metrics

The segmentation performance of the transformer enhanced U-Net and contrast-optimized PDN models is assessed using three key metrics. These metrics were specifically chosen because they directly measure the degree of spatial overlap and boundary accuracy. The dice similar coefficient quantifies the overlap between the predicted region $S_{\text{pred}}$ and the predicted mask $S_{\mathrm{gt}}$ where a higher value (closer to 1) indicates better segmentation accuracy as shown in Equation 22. The dice loss defined as the negative dice similar coefficient is minimized during model training to maximize the agreement between predicted tumour region and predicted masks is expressed in Equation 23. The intersection over union also known as the jaccard index which measures the ratio of intersection to union of $S_{\text{pred}}$ and $S_{\mathrm{gt}}$ offering a robust evaluation by considering both false positives and false negatives as shown in Equation 24. These metrics measure the accuracy of comparison between the predicted mask and the actual tumor region. Dice loss assesses overlap accuracy whereas intersection over union considers errors from both false positives and false negatives. Collectively, they provide an in-depth evaluation of segmentation efficacy.

$$\;\mathrm{DSC}=\frac{2\cdot |S_{\text{pred}}\cap S_{\mathrm{gt}}|+\varepsilon \;}{|S_{\text{pred}}|+|S_{\mathrm{gt}}|+\varepsilon \;}$$ (22)

$$\text{Dice Loss}=-\mathrm{DSC}=\frac{2\cdot |S_{\text{pred}}\cap S_{\mathrm{gt}}|+\varepsilon \;}{|S_{\text{pred}}|+|S_{\mathrm{gt}}|+\varepsilon \;}$$ (23)

$$\mathrm{IoU}=\frac{|S_{\text{pred}}\cap S_{\mathrm{gt}}|+\varepsilon \;}{|S_{\text{pred}}\cup S_{\mathrm{gt}}|+\varepsilon \;\;}$$ (24)

**Figure 12:**
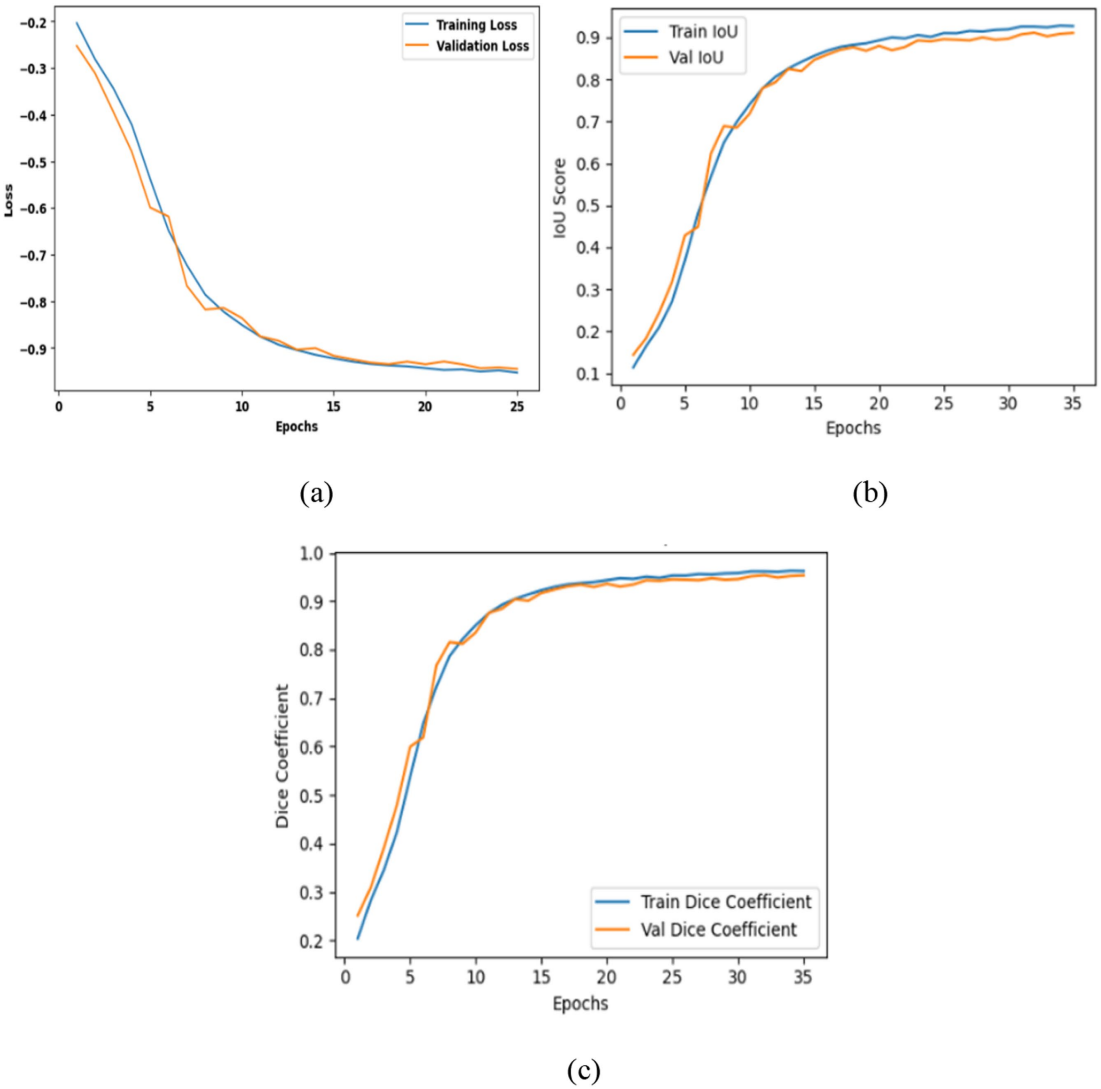
Evaluation of the enhanced U-Net model: **(a)** Training and validation loss; **(b)** intersection over union; **(c)** Dice coefficient.

Table 6 presents the segmentation performance of the three models by using averaged data from multiple seeds expressed as mean ± standard deviation and along with their 95% confidence intervals. This provides a more precise and statistically validated comparison. The transformer augmented U-Net attained an IoU of 0.9107 and a dice coefficient of 0.9532 representing precise spatial reconstruction. This architecture includes a vision transformer module into the traditional U-Net framework integration an encoder decoder structure with convolutional layers, multi-head self-attention and skip connections to collect both local and global contextual information. Figure 12 shows the training curves and ROC analysis indicating the model convergence and strong segmentation performance.

**Table 6:** Performance evaluation of segmentation models.

Model	Structure	DICE (mean ± SD) [95% CI]	IOU (mean ± SD) [95% CI]	HD95 (mean ± SD) [95% CI]	ASSD (mean ± SD) [95% CI]	LOSS (mean ± SD) [95% CI]
VHU_net	Kidney	0.9532 ± 0.0134 [0.9269–0.9795]	0.9107 ± 0.0242 [0.8633–0.9581]	0.2692 ± 0.4436 [0.0000–1.1387]	0.0743 ± 0.0384 [0.0000–0.1496]	0.0468 ± 0.0133 [0.0207–0.0729]
	Tumor	1.0000 ± 0.0000 [1.0000–1.0000]	1.0000 ± 0.0000 [1.0000–1.0000]	–	–	–
ConD-PDN	Kidney	0.9629 ± 0.0136 [0.9362–0.9896]	0.9285 ± 0.0249 [0.8797–0.9773]	0.2692 ± 0.4436 [0.0000–1.1387]	0.0624 ± 0.0287 [0.0061–0.1187]	0.0371 ± 0.0136 [0.0104–0.0638]
	Tumor	1.0000 ± 0.0000 [1.0000–1.0000]	1.0000 ± 0.0000 [1.0000–1.0000]	–	–	–
Fuse_models	Kidney	0.9712 ± 0.0088 [0.95395–0.98845]	0.9441 ± 0.0164 [0.91196–0.97624]	0.0769 ± 0.2665 [0.0000–0.5992]	0.0504 ± 0.0186 [0.01394–0.08686]	0.0288 ± 0.0088 [0.01155–0.04605]
	Tumor	1.0000 ± 0.0000 [1.0000–1.0000]	1.0000 ± 0.0000 [1.0000–1.0000]	–	–	–

**Figure 13:**
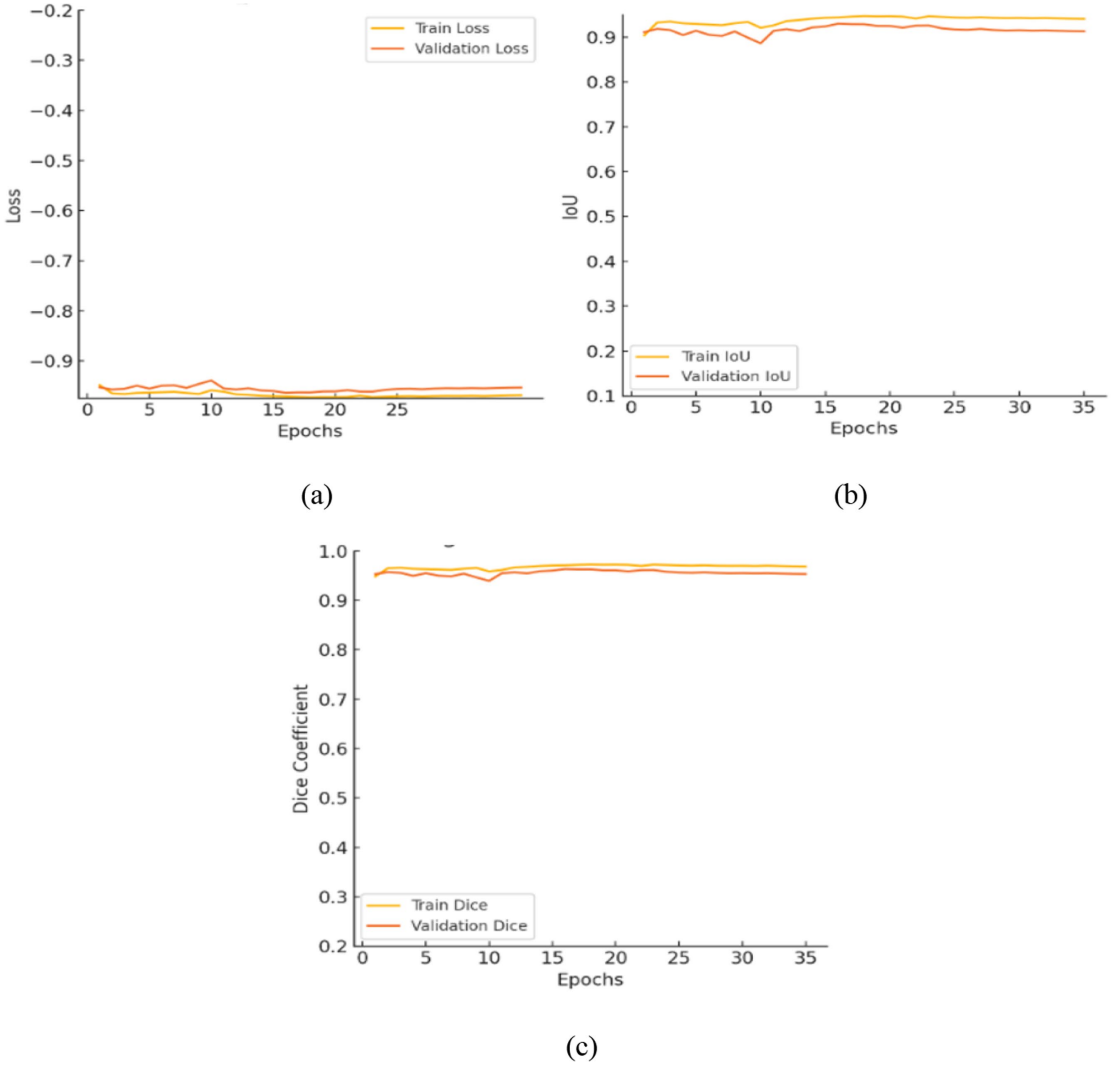
Analysis of contrast optimized PDN model: **(a)** Training and validation loss, **(b)** intersection over union, **(c)** dice coefficient.

The contrast optimized PDN improves segmentation by improving contrast facilitating exact characterization of structural boundaries. The design includes convolutional layers with contrast based feature improvement, batch normalization, multiscale pooling and non-linear activations to improve segmentation accuracy. In the test dataset, model attained an IoU of 0.9285 and a dice coefficient of 0.9629 indicating enhanced accuracy and less segmentation error. Figure 13 shows the training curves and ROC analysis validating consistent learning and improved boundary recognition.

**Figure 14:**
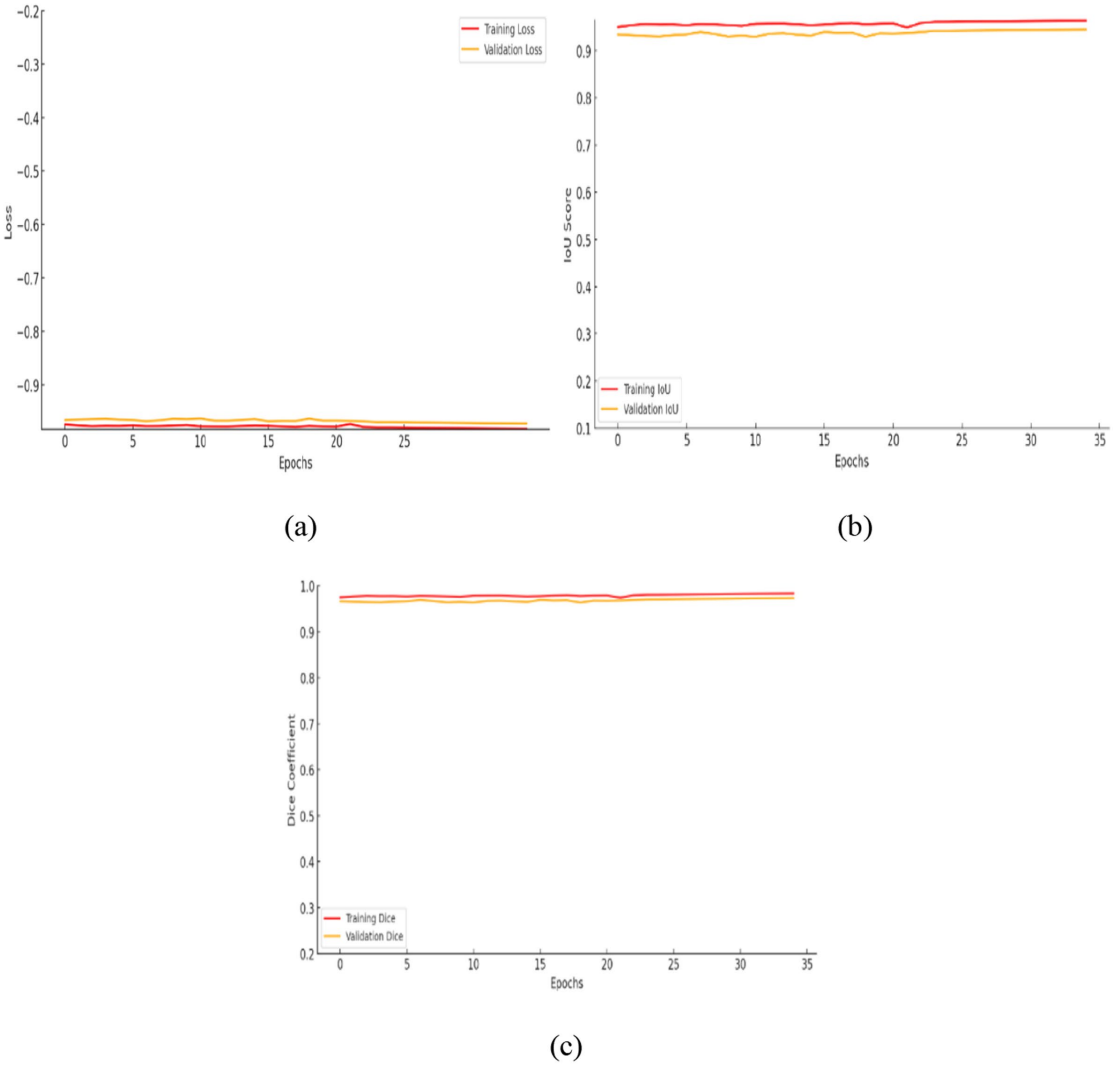
Analysis of proposed VHUCS-Net model: **(a)** Training and validation loss; **(b)** intersection over union; **(c)** dice coefficient.

The proposed VHUCS-Net a hybrid of the transformer enhanced U-Net and contrast optimized PDN combines global context modelling with contrast driven feature refining to attain increased segmentation performance. The hybrid model attained an IoU of 0.9441 and a dice coefficient of 0.9712 outperforming the performance of the individual models and showing that the fusion of features improves both segmentation precision and spatial overlap. Figure 14 illustrates the performance curves and ROC analysis which highlight the enhancements hybrid framework.

The confusion matrix provides a detailed analysis of predictions by class and displays patterns of misclassification as the associated heatmap visually highlights error distribution and performance at the class level. Table 7 presents the confusion matrices with their corresponding heatmaps facilitating a detailed evaluation of the model performance.

**Table 7:** Confusion matrices and heatmaps.

Confusion matrix	HeatMap
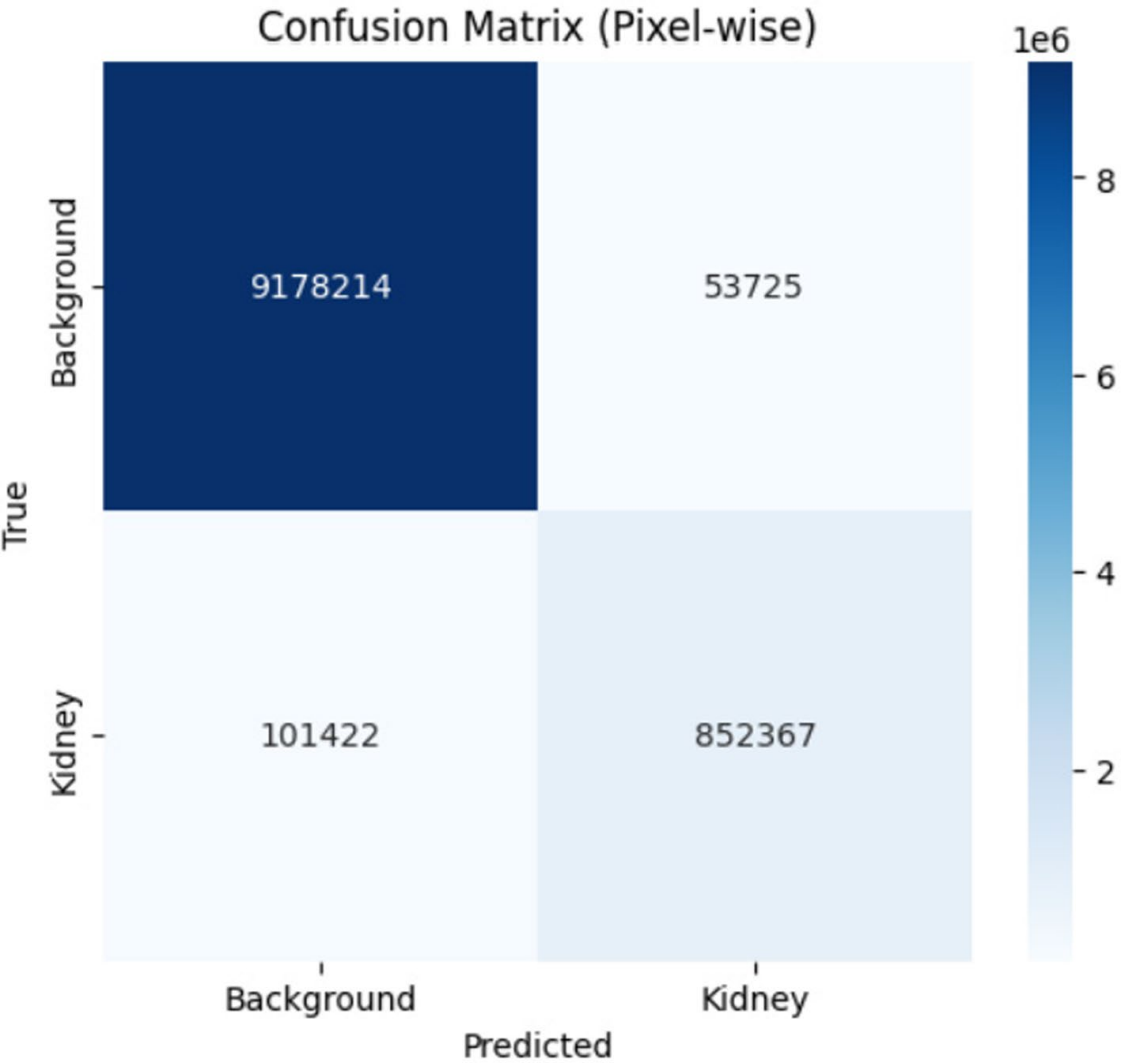	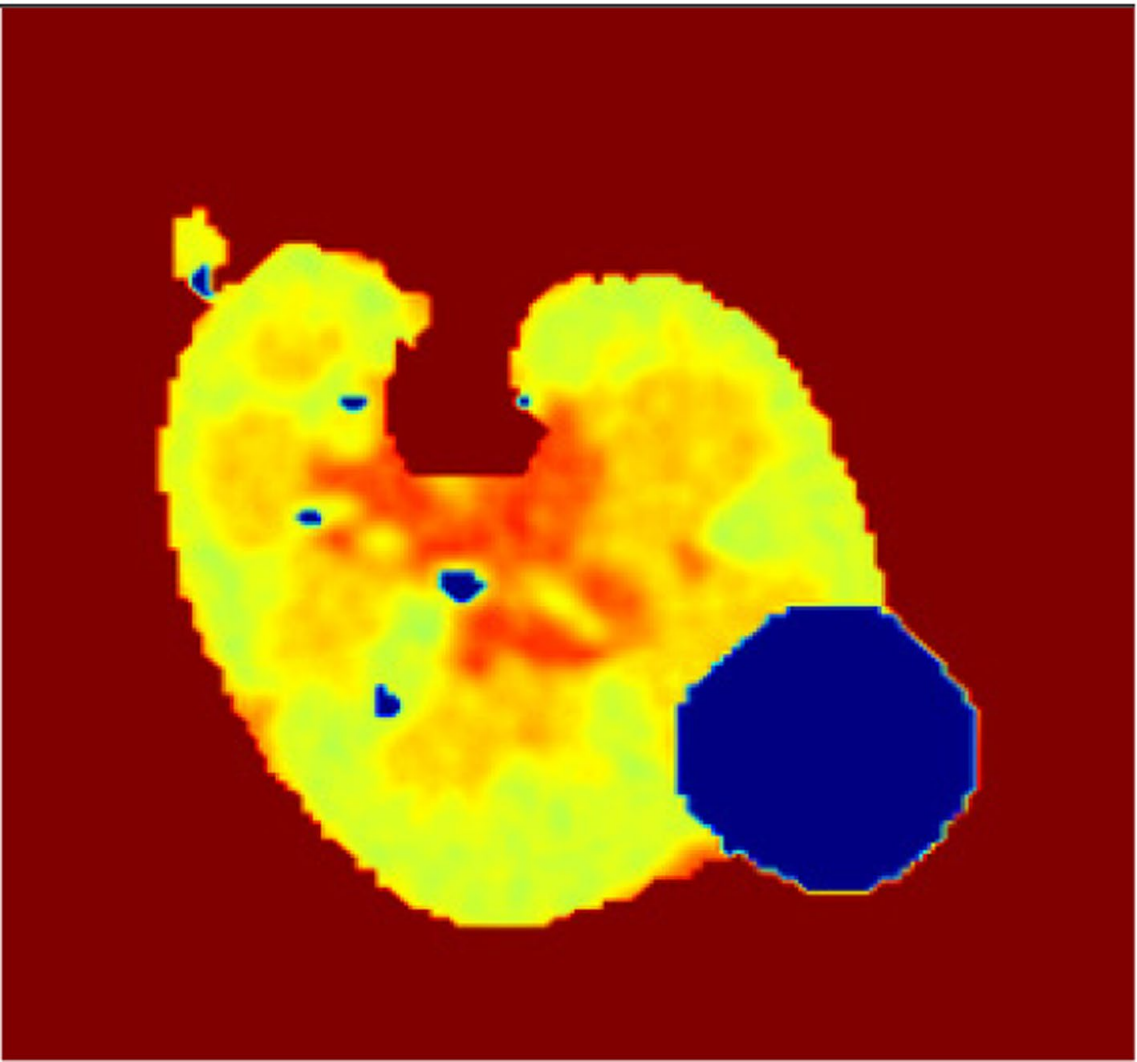

### 4.7 Performance analysis of model output

**Figure 15:**
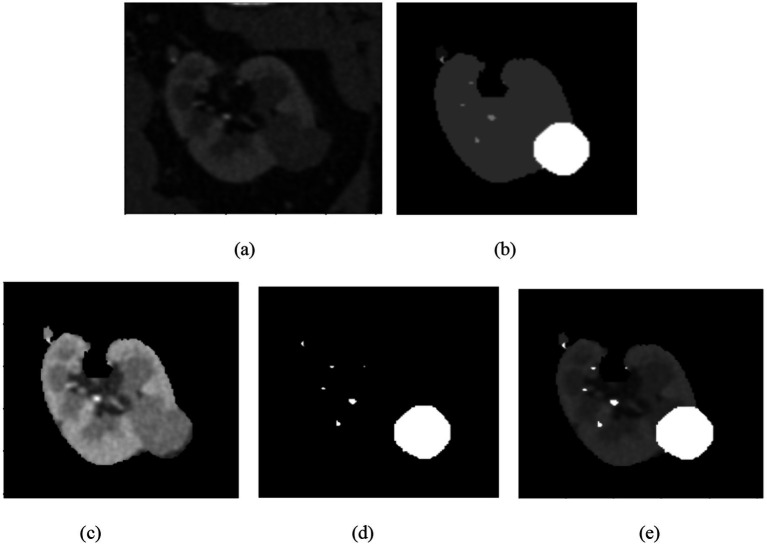
Visualization of the kidney segmentation process: **(a)** Original kidney image, **(b)** mask image, **(c)** segmented kidney region, **(d)** segmented tumor mask, **(e)** fused kidney tumor image.

**Figure 16:**
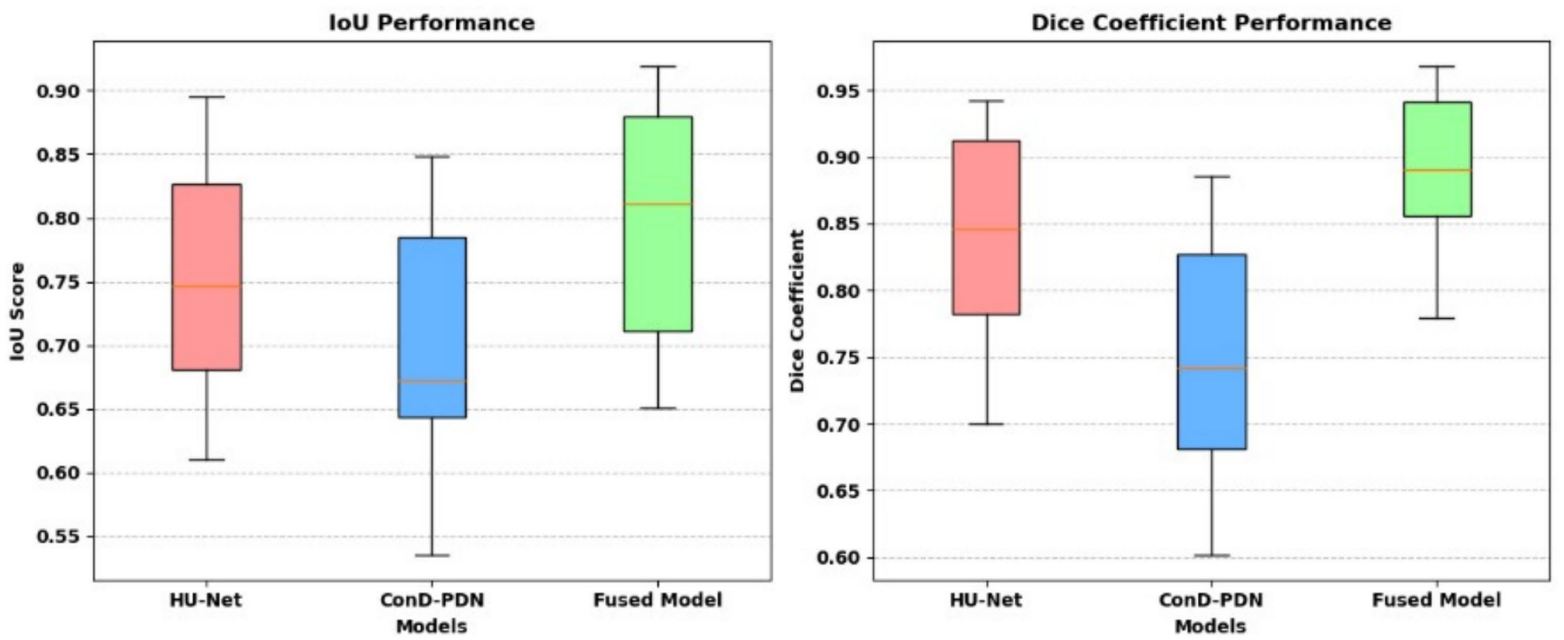
IoU and dice coefficient performance comparison.

The segmentation phase performs in two tracks in track 1 employs a transformer enhanced U-Net model while track 2 uses the contrast optimized PDN model. The input to the transformer enhanced U-Net model contains a sliced kidney image containing masses. This model incorporates a standard U-Net model with a ViT layer and a HRNet as decoder. The ViT component captures long range dependencies and global context and the HRNet preserves detailed spatial information (Gong and Kan, 2021). The contrast optimized PDN model is specifically designed to segment the kidney mass from the masked kidney image. It employs multi scale max pooling for capturing both fine and coarse details also, the use of separableconv2D reduces computational difficulty while maintaining accuracy. Figure 15 illustrates the kidney segmentation approach utilizing the suggested dual track framework. The original kidney image is shown in (a) followed with the corresponding mask in (b) track 1 the transformer enhanced U-Net precisely segments the kidney region as illustrated in (c) whereas track 2 the contrast optimized PDN segments the renal tumor presented in (d). The outputs from both tracks are later fused in the fusion stage resulting in the final fused kidney tumor segmentation in (e). The resulting combination improves boundary accuracy, incorporates structural variations and provides efficient multi-scale feature integration (MRFA-Net, n.d.) leading to dependable and precise kidney mass identification. The fusion of the transformer enhanced U-Net and contrast optimized PDN models achieves higher IoU and dice coefficient performance as shown in Figure 16. This performance improvement explains the corresponding benefits of the two frameworks includes superior spatial detail preservation from the transformer enhanced U-Net and better localized feature extraction from the contrast optimized PDN. The model utilizes multi-scale information to enhance boundary precision and robustness to morphological variability for medical image analysis.

### 4.8 VHUCS-net validation on publicly accessible datasets

To evaluate the generalizability of the proposed VHUCS-Net model experiments were performed on various publically available medical image segmentation datasets which includes the Skin Cancer MNIST: HAM10000 dataset, the Blood Cell Segmentation Dataset and the KiTS23 kidney tumor segmentation dataset. Images for the skin lesion segmentation analysis were obtained from the Skin Cancer MNIST: HAM10000 dataset (Mader, 2018) and the corresponding lesion masks were acquired from the HAM10000 Lesion Segmentations dataset (Mader, 2018). The HAM10000 dataset (Human Against Machine with 10,000 training photos) consists of 10,015 skin lesion images obtained from different people and imaging techniques. The Blood Cell Segmentation Dataset (BCCD) (Deponker et al., 2023) contains pixel-level annotations along with consistent image-mask pairings. Out of the 1,328 image and mask pairs, a selected subset of 1,169 pairs were used for quantitative studies whereas the remaining pairs are provided with the corresponding script for transparency but excluded from training and evaluation. The KiTS23 dataset (Kumar, n.d.) which includes annotated axial CT slices for kidney tumor segmentation has been evaluated with data augmentation applied to the training set resulting in 39,080 augmented image and mask pairs. Validation and testing were performed on non-augmented data consisting of 3,965 validation pairs and 3,850 test pairs using patient-wise partitioning to prevent data leakage. All datasets were divided into training, validation and test partitioned outlined in Table 8.

**Table 8:** Dataset partitioning for proposed VHUCS-Net model validation using additional open-source datasets.

Dataset	Training	Validation	Test	Total
Skin lesion	8,012	1,001	1,002	10,015
Blood cell	935	117	117	1,169
KiTS23	39,080	3,965	3,850	46,895

**Figure 17:**
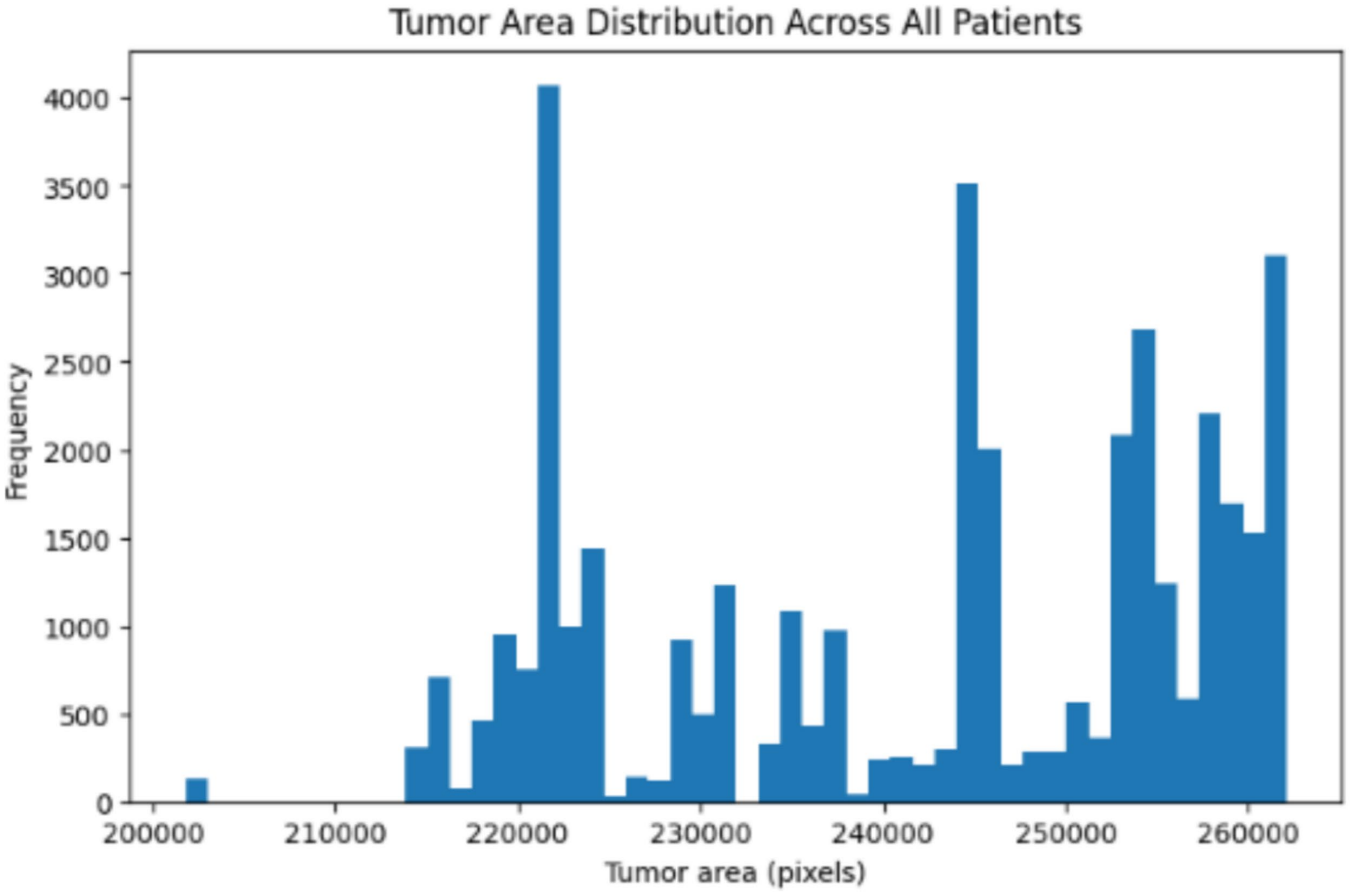
Tumour area distribution.

Figure 17 show the tumour area distribution across all patient. The proposed VHUCS-Net model was trained and validated on additional datasets with same hyper parameter values used in the kidney disease segmentation challenge. The evaluation of model performance was done using the dice coefficient and IoU as illustrated in Table 9. Table 10 shows the runtime and resource utilization of VHUCS-Net on the KiTS23 dataset. Segmentation results were generated where affected regions are highlighted clearly illustrate the model efficiency in exactly determining and differentiating target areas. These visualizations provide a direct comparison of VHUCS-Net segmentation efficacy across the Kidney, HAM10000 (skin lesion), Blood Cell datasets and KiTS23 with quantitative results presented in Table 11.

**Table 9:** Performance comparison of VHUCS-Net on different datasets.

Dataset	Loss	IoU (mean ± SD, 95% CI)	Dice (mean ± SD, 95% CI)	HD95 (mean ± SD, 95% CI) [mm]	ASSD (mean ± SD, 95% CI) [mm]	Per-Volume Latency (GPU)	Peak Memory Usage (GPU)
Kidney	0.0288	0.9441 ± 0.0062	0.9712 ± 0.0034	0.077 ± 0.267	0.050 ± 0.019	0.33 s	~1.1–1.2 GB VRAM
Skin lesion	0.0881	0.8405 ± 0.0081	0.9119 ± 0.0068	3.94 ± 0.72	1.42 ± 0.18	0.33 s	~1.0–1.1 GB VRAM
Blood Cell	0.0360	0.9306 ± 0.0070	0.9640 ± 0.0045	2.11 ± 0.55	0.77 ± 0.11	0.33 s	~0.9–1.0 GB VRAM
KiTS23	0.0432	0.8845 ± 0.0717	0.9370 ± 0.0442	1.0504 ± 2.4964	0.1980 ± 0.3115	0.57 s	~1.28 GB VRAM

**Table 10:** Runtime and resource usage on KiTS23.

Dataset	Patients	Median slices/volume (IQR)	Time per slice (s)	Time per volume (s)	Hardware	Peak memory
KiTS23	100	390.8 (390.8–390.8)	0.001463	0.572	GPU (Tesla P100, fp32)	1.28 GB VRAM

**Table 11:** Comparison of VHUCS-Net segmentation results with contour overlay across different datasets.

Dataset			
	Kidney	Skin lesion	Blood cell
Image segmentation visualization	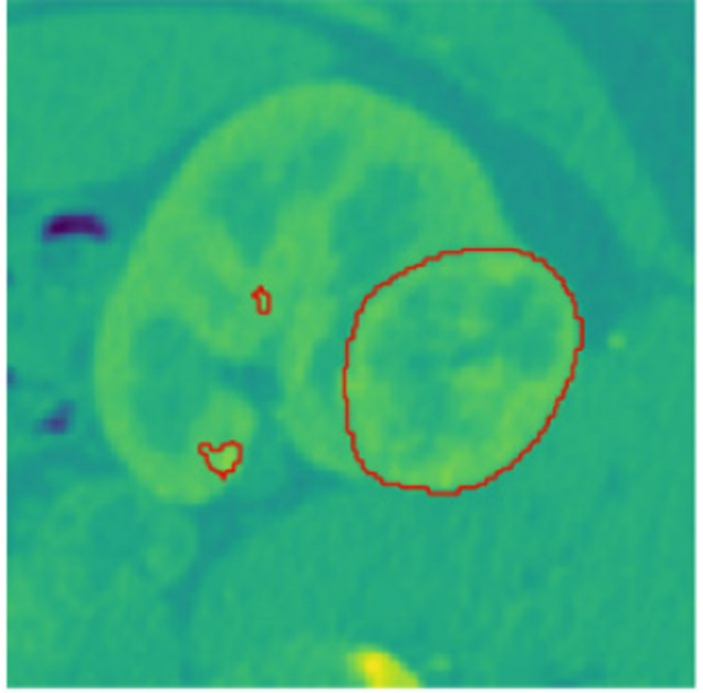	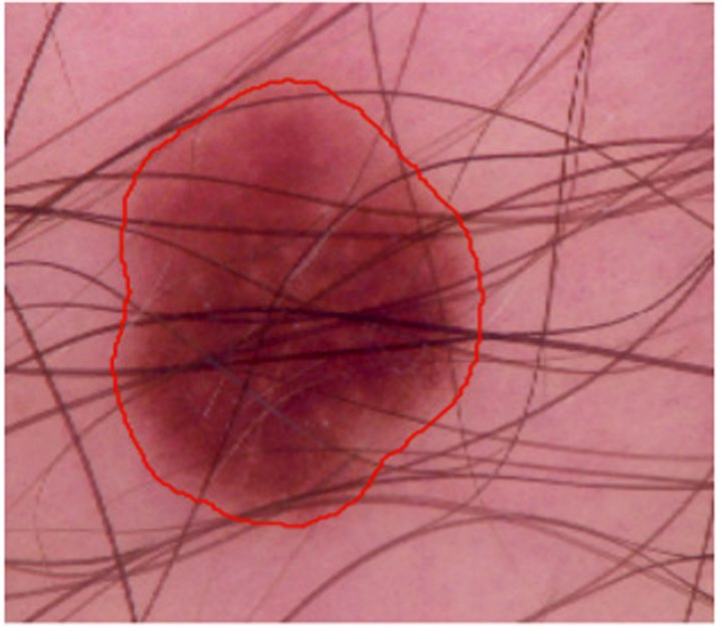	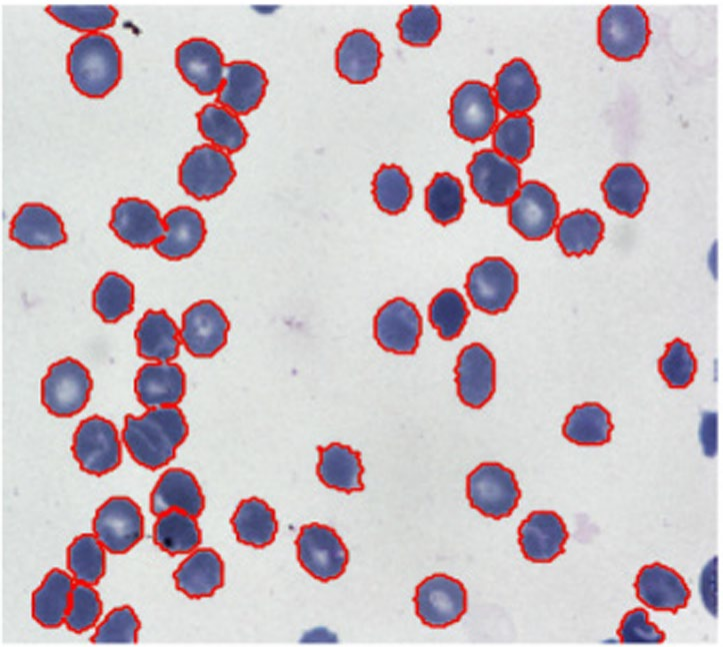

### 4.9 Ablation study

An ablation study has been conducted for systematic evaluation of the contribution of various modules within the proposed architecture by selectively adjusting the model structure. The evaluation utilized key performance metrics including the dice coefficient (Eapen et al., 2015), IoU, loss, (Eapen et al., 2016) total parameter counts and model size.

#### 4.9.1 Performance analysis transformer enhanced U-net with ViT layer

**Figure 18:**
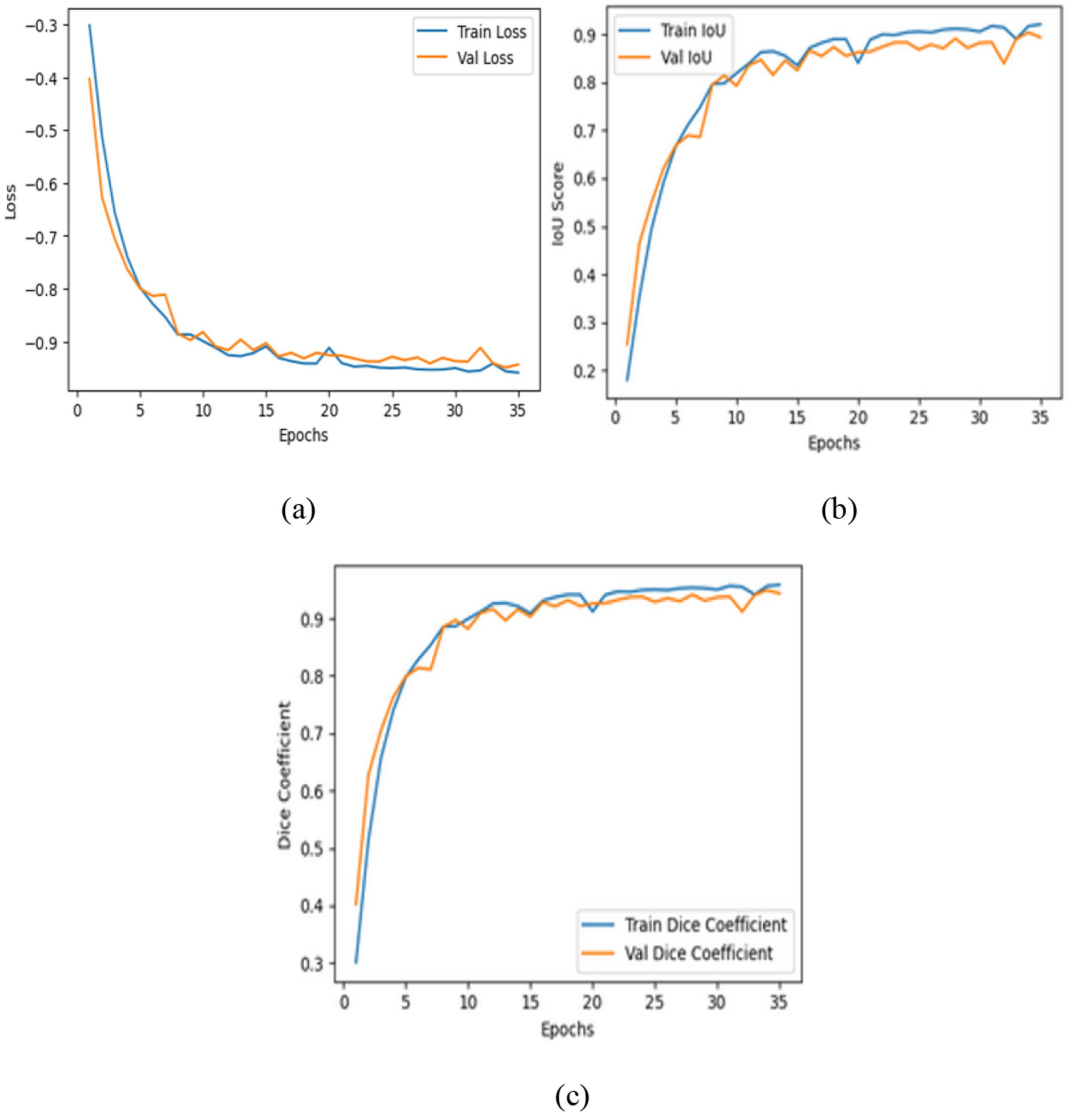
Performance analysis of transformer enhanced U-Net with ViT layer: **(a)** Loss, **(b)** IoU, **(c)** dice coefficient.

This implementation assesses the effects of incorporating a ViT attention layer which enhances global context modeling and improves feature extraction. The model attains a dice coefficient of 0.9436 and an IoU of 0.8937 enabled by HRNet robust spatial preservation. With 7.78 million parameters and a size of 29.69 MB it exhibits modest complexity while achieving high segmentation performance as shown in Figures 18a–c capacity.

#### 4.9.2 Performance analysis transformer enhanced U-net with HRNet layer

**Figure 19:**
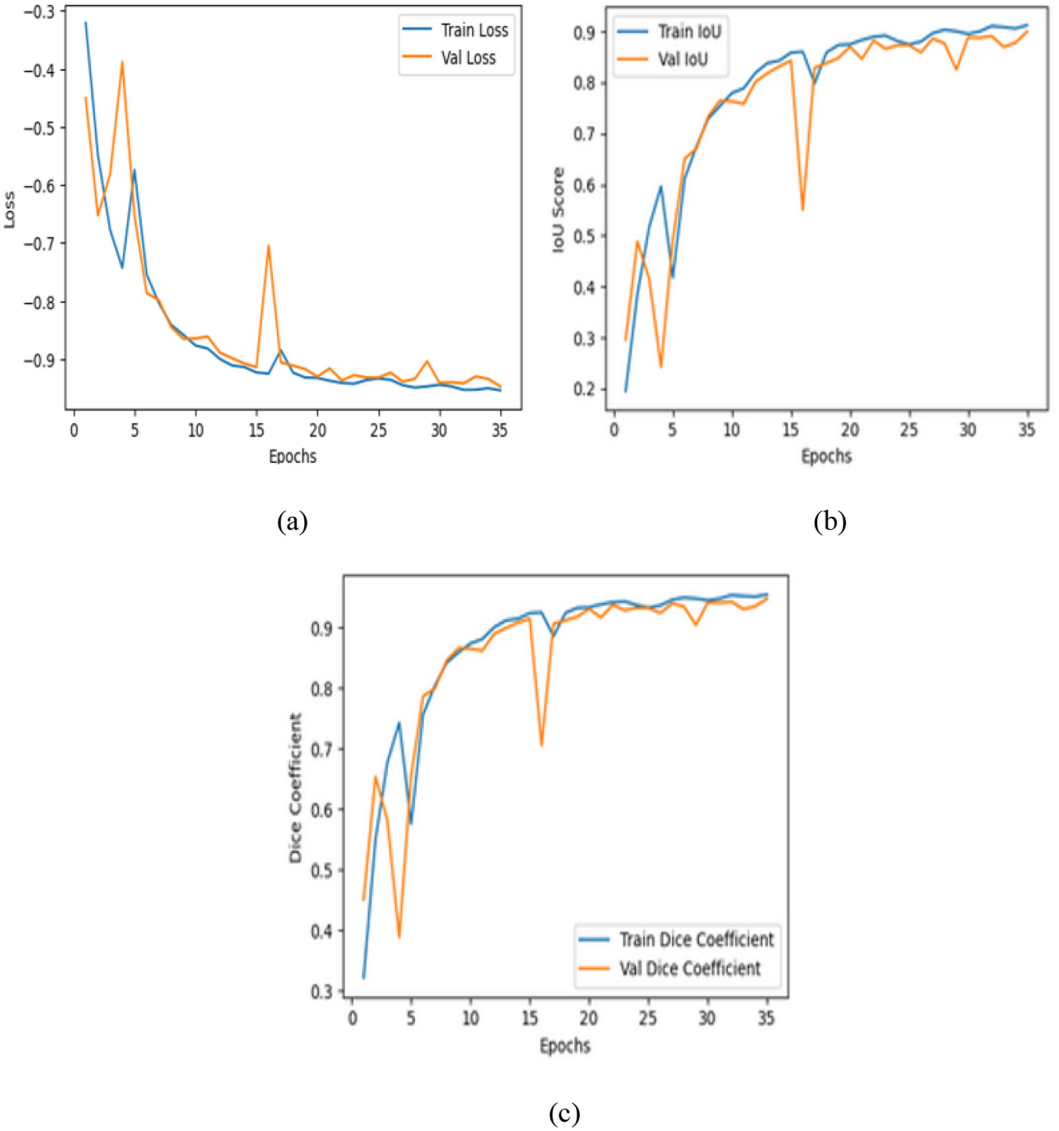
Performance analysis of transformer enhanced U-Net with HRNet layer: **(a)** Training and validation loss; **(b)** intersection of union; **(c)** Dice coefficient.

This configuration uses HRNet to maintain high-resolution features and integrate multi-scale information enhancing structural detail and boundary localization. It attains a dice coefficient of 0.9472 and an IoU of 0.9001 including exactly 196,916 parameters and a size of 0.75 MB indicating of robust accuracy and efficiency. Figures 19a–c shows the curves for loss, intersection over union and dice coefficient.

#### 4.9.3 Performance analysis of contrast optimized PDN model

**Figure 20:**
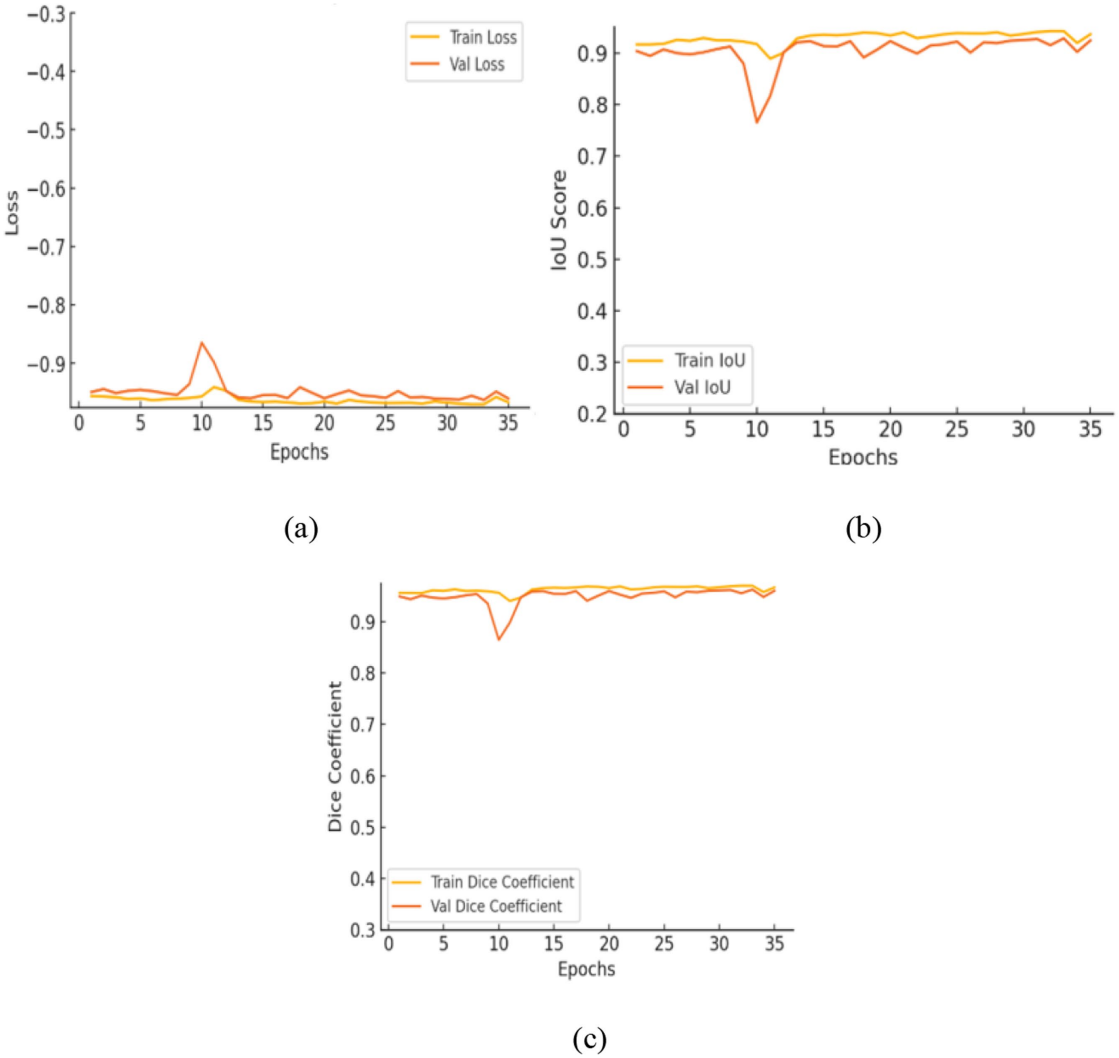
Performance analysis of contrast optimized PDN model: **(a)** Loss, **(b)** IoU, **(c)** dice coefficient.

This implementation evaluates the contrast-optimized PDN model, which improves border detection via superior contrast management and enhanced edge processing. It attains a dice coefficient of 0.9605 and an IoU of 0.9245 indicating robust segmentation consistency. With 3.37 million parameters and a size of 12.86 MB it is both lightweight and efficient as shown by the loss, IoU and dice curves illustrated in Figures 20a–c.

The ablation study validates that each design component distinctly, ViT attention layer is essential for acquiring global contextual information allowing the model to analyse long-range dependencies more effectively. The HRNet decoder is essential for maintaining high-resolution spatial features thus providing an accurate representation. The contrast optimized PDN module specifically with its multiscale pooling technique significantly improves mass localization and sharpens borders. The fusion of these components generates excellent performance, showing the significance of each individual module for efficient kidney mass segmentation. Table 12 summarizes the ablation study where all architectural variants including ViT-only, HRNet-only and the combined ViT + HRNet modules are evaluated. Parameter count and model size are also compared to offer a comprehensive understanding of computational complexity and performance.

**Table 12:** Ablation study of the model components.

Model	Loss (mean ± SD) [95% CI]	IoU (mean ± SD) [95% CI]	Dice (mean ± SD) [95% CI]	Total Params	Model size
U-Net with ViT	0.0528 ± 0.0026 [0.0477–0.0579]	0.9001 ± 0.0093 [0.8819–0.9183]	0.9472 ± 0.0068 [0.9340–0.9603]	196,916	0.75 MB
U-Net with HRNet	0.0564 ± 0.0031 [0.0502–0.0626]	0.8937 ± 0.0112 [0.8719–0.9155]	0.9436 ± 0.0074 [0.9292–0.9580]	196,916	0.75 MB
U-Net with ViT + HRNet	0.0468 ± 0.0022 [0.0422–0.0514]	0.9107 ± 0.0086 [0.8931–0.9283]	0.9532 ± 0.0059 [0.9417–0.9647]	7,781,761	29.69 MB
Contrast-optimized PDN	0.0395 ± 0.0018 [0.0359–0.0431]	0.9245 ± 0.0074 [0.9100–0.9390]	0.9605 ± 0.0048 [0.9512–0.9697]	3,370,000	19.70 MB
Proposed VHUCS-Net	0.0288 ± 0.0011 [0.0266–0.0310]	0.9441 ± 0.0062 [0.9318–0.9564]	0.9712 ± 0.0034 [0.9646–0.9778]	32,624,261	124.45 MB

### 4.10 Comparison of proposed model with state of architecture

Various kidney segmentation methods have been studied across different datasets demonstrating significant performance improvement. Kittipongdaja and Siriborvornratanakul (2022) utilised 2.5D ResU-Net and 2.5D DenseU-Net on the KiTS19 and Thai Patient Datasets achieving dice scores of 0.95 and 0.87. Hatsutani (2023) established a PDN on KiTS19 achieving a dice score of 0.615 and a sensitivity of 0.721, effectively recognizing protruding tumour areas. Bolocan et al. (2023) employed U-Net and ResNet101 on private DICOM images attaining dice scores of 0.675 for tumours and 0.84 for kidneys. Swain et al. (2024) used YOLOv8 and Mask R-CNN on the HuBMAP dataset indicating a precision of 0.97, recall of 0.85 and mAP50 of 0.93. Oghli et al. (2024) implemented Fast U-Net++ on the Open Kidney Dataset achieving sagittal and axial dice scores of 0.97 and 0.95, respectively. Zhao et al. (2020) developed a multi-scale supervised 3D U-Net on KiTS19 achieving segmentation performance with dice scores of 0.969 for kidneys and 0.805 for tumours. Zhao et al. (2023) proposed a cascade 3D U-Net and ResNet on KiTS21 attaining accurate kidney mass segmentation with dice scores of 0.99 for kidneys and 0.75–0.83 for kidney masses. Conze et al. (2024) evaluated various models including v19p U-Net, Trans U-Net, MedT, Segmenter, and Swin U-NetV2 on the Genkyst dataset, with SwinUNetV2 outperforming the other models in complex segmentation tasks achieving a dice score of 0.934. Hsiao et al. (2022a) combined EfficientNet-B5 and FPN on KiTS19 and 3D-IRCAD-01, increasing segmentation efficacy with a dice score of 0.969. Hsiao et al. (2022b) utilised ResNet-41 and EfficientNet on KiTS19 enhancing segmentation precision by preprocessing, resulting in dice scores of 0.9648 for kidneys and 0.7294 for tumours. Jariwala et al. (2024) integrated U-Net and DeepLabv3 + on KiTS23 optimizing segmentation precision with a dice score of 0.94. Causey et al. (2021) deployed an ensemble of U-Net models with post-processing on KiTS19 improving segmentation precision it achieved 0.9470 dice score for kidneys and 0.6099 for tumours. Proposed model, which combines transformer enhanced U-Net model and contrast-optimized PDN model on the kidney segmentation dataset attained enhanced segmentation accuracy with improved kidney mass localization and boundary precision demonstrated by a loss of 0.0288, an IoU of 0.944 and a dice coefficient of 0.9712 as presented in Table 13. The segmentation performance of VHUCS-Net with standard baseline models (U-Net, UNet++, MobileNetV2) re-run on the kidney segmentation dataset using the same metrics is shown in Table 14.

**Table 13:** Performance comparison with other state of art methods.

Ref	Dataset	Methodology	Evaluation metrics	Key characteristic
Kittipongdaja and Siriborvornratanakul (2022)	KiTS19 and Thai patient	2.5D ResU-Net, 2.5D DenseU-Net	Dice: 0.95 (KiTS19), 0.87 (Thai)	Achieved high segmentation accuracy across dataset
Hatsutani (2023)	KiTS19	Protuberance Detection Network	Dice: 0.615, sensitivity: 0.721	Accurate in identifying protruding tumor regions
Bolocan et al. (2023)	Private (raw DICOM images)	U-Net, ResNet101	Dice: 0.675 (Tumour), 0.84 (kidney)	U-Net provide precise segmentation outcomes.
Swain et al. (2024)	HuBMAP	YOLOv8, Mask R-CNN	Precision: 0.97, recall: 0.85, mAP50: 0.93	YOLOv8 provides higher segmentation accuracy and efficiency.
Oghli et al. (2024)	Open kidney data set	Fast U-Net++	Dice: 0.97 (sagittal), 0.95 (axial)	Exactly predicts kidney shape and volume
Zhao et al. (2020)	KiTS19	Multi-scale supervised 3D U-Net	Dice: 0.969 (kidney), 0.805 (tumour)	Efficient segmentation with multi-scale supervision
Zhao et al. (2023)	KiTS21	Cascading 3D U-Net, ResNet	Dice: 0.99 (kidney), 0.75–0.83 (kidney mass)	Attains accurate segmentation of kidney boundaries
Conze et al. (2024)	Genkyst	v19p U-Net, Trans U-Net, MedT, Segmenter, Swin U-NetV2	Dice: 0.934 (both organ), 0.934 (independent & dual task)	Swin U-NetV2 provides better results in complex segmentation cases.
Hsiao et al. (2022a)	KiTS19, 3D-IRCAD-01	EfficientNet-B5, FPN	Dice: 0.969 (KiTS19)	FPN optimises segmentation efficiency and enhancement.
Hsiao et al. (2022b)	KiTS19	ResNet-41 and EfficientNet	Dice: 0.9648 (kidney), 0.7294 (tumour)	Pre-processing methods enhance segmentation accuracy.
Jariwala et al. (2024)	KiTS23	U-Net and DeepLabv3+,	Dice: 0.94	DeepLabv3 + enhances segmentation accuracy
Causey et al. (2021)	KiTS19	Ensemble of U-Net models with post pre-processing	Dice: 0.9470 (kidney), 0.6099 (tumour)	Post-processing enhances segmentation accuracy
Proposed VHUCS-Net	Kidney Segmentation Dataset	Transformer-enhanced U-Net model and contrast-optimized Protuberance Detection Network (PDN) model	Loss of 0.0288, IoU: 0.9441, dice coefficient: 0.9712	Achieves high segmentation accuracy with enhanced boundary precision and optimized kidney mass localization.

**Table 14:** Segmentation performance of VHUCS-Net and baseline models with kidney segmentation dataset.

Method	Dice (mean ± SD) [95% CI]	IoU (mean ± SD) [95% CI]
U-Net	0.8320 ± 0.0041 [0.8240–0.8400]	0.7468 ± 0.0050 [0.7370–0.7566]
UNet++	0.8594 ± 0.0036 [0.8523–0.8665]	0.7846 ± 0.0044 [0.7759–0.7933]
MobileNetV2	0.8057 ± 0.0050 [0.7959–0.8155]	0.6995 ± 0.0060 [0.6878–0.7112]
DeepLabV3 + (MobileNetV2 backbone)	0.8673 ± 0.0038 [0.8600–0.8746]	0.7902 ± 0.0042 [0.7819–0.7985]
VHUCS-Net (proposed)	0.9712 ± 0.0034 [0.9646–0.9778]	0.9441 ± 0.0062 [0.9318–0.9564]

## 5 Conclusion and future work

Kidney masses exhibit significant variation in size, shape and texture across individuals making it essential for segmentation models to achieve both high accuracy and adaptability. The proposed VHUCS-Net model statements this challenge using a dual-track architecture such as transformer enhanced U-Net model in track 1 and the contrast optimized PDN model in track 2. The transformer enhanced U-Net model features an encoder that combines ViT attention with HRNet with the standard U-Net architecture. The ViT attention mechanism enhances global feature representation by capturing long-range dependencies, hence improving the difference between kidney structures and surrounding tissues. HRNet maintains detailed spatial information important for efficient segmentation while the U-Net decoder preserve spatial information through skip connections, enhancing boundaries and enhancing localization of the kidney region. Thus, the transformer-enhanced U-Net effectively segments the kidney region from the neighbouring tissues attaining an IoU of 0.9107 and a dice value of 0.9532 indicating robust feature extraction and accurate segmentation. The contrast optimized PDN model simultaneously highlighting mass regions inside the kidney. It employs multi-scale pooling to extract features with various sizes and utilize SeparableConv2D layers to enhance boundaries effectively. The implementation of further batch normalization and feature fusion enhances model accuracy and adaptability providing the contrast-optimized PDN more effective for kidney mass segmentation. This is shown in its performance attaining an IoU of 0.9285 and a dice coefficient of 0.9629 showing accuracy and consistency. The fusion of these two models in the final VHUCS-Net architecture incorporates their respective strengths with global context integration accurate spatial detail preservation and exact mass localization. In the Kidney Segmentation Dataset VHUCS-Net attained an IoU of 0.9441 and a dice coefficient of 0.9712. The results indicate that the fusion of both models provides a highly accurate and reliable method for renal mass segmentation. Future study will explore integrating 3D attention modules and self-supervised pretraining to further strengthen VHUCS-Net multi-organ segmentation capabilities. Aim to develop the model to larger and more diverse datasets to optimize its usefulness across various clinical contexts. Clinical deployment studies will also be carried out to assess its efficacy and simplify the integration of real-world workflow.

## Data Availability

The original contributions presented in the study are included in the article/supplementary material, further inquiries can be directed to the corresponding authors.
